# Efficacy and safety of plant-derived bioactive compounds in the treatment of psoriasis vulgaris: a systematic review and network meta-analysis

**DOI:** 10.3389/fmed.2026.1844822

**Published:** 2026-06-22

**Authors:** Yue Du, Shouxu Zhang, Tian Lv, Xiaoxuan Chen, Lingling Yuan, Haomin Zhang, Guangshan Chen, Weiling Chen, Lingling Li

**Affiliations:** 1 Department of Dermatology, Dongzhimen Hospital, Beijing University of Chinese Medicine, Beijing, China; 2 Beijing University of Chinese Medicine, Beijing, China; 3 Department of Chinese Medicine and Rehabilitation, Beijing Health Vocational College, Beijing, China

**Keywords:** network meta-analysis, oral treatment, plant-derived bioactive compounds, psoriasis vulgaris, randomized controlled trials

## Abstract

**Background:**

Long-term use of conventional therapy (CT) for psoriasis vulgaris (PV) is often constrained by adverse effects and costs. While oral plant-derived bioactive compounds show clinical promise as adjunctive treatments, the relative efficacy and safety of these compounds and their combination regimens remain poorly defined due to a lack of head-to-head comparisons. This systematic review and network meta-analysis aimed to compare and rank the efficacy and safety of different oral plant-derived bioactive compounds and their combination regimens for PV.

**Methods:**

Randomized controlled trials investigating the effects of plant-derived bioactive compounds, such as compound glycyrrhizin (CG), tripterygium glycosides (TG), and total glucosides of paeony (TGP), on PV were retrieved from Chinese and English databases via computer searches. The main outcome measures included the Psoriasis Area and Severity Index (PASI), CD4^+^/CD8^+^ ratio, IL-23, IL-17, and TNF-*α*. Network meta-analysis was performed using Stata 16.0 software.

**Results:**

We included 84 randomized controlled trials comprising 7,544 patients across 15 treatment regimens. Most combination therapies significantly outperformed monotherapy in PASI reduction. TG combined with topical therapy (TOP) produced the greatest reduction in PASI scores [mean difference (MD) = −13.96, 95% confidence interval (CI) (−16.88, −11.03)], with the highest surface under the cumulative ranking curve (SUCRA) value of 99.80%. Individual compounds showed distinct advantages for specific therapeutic targets. CG + TOP proved most effective for reducing IL-23 levels (SUCRA = 97.30%), while CG + NB-UVB achieved the greatest IL-17 reduction (SUCRA = 91.20%). CG + CT best restored CD4^+^/CD8^+^ ratio balance (SUCRA = 94.70%), and TGP + TOP led all regimens in TNF-*α* suppression (SUCRA = 92.40%). Adverse events were predominantly mild, consisting of gastrointestinal symptoms or skin dryness. No serious or irreversible events were recorded.

**Conclusion:**

Combining plant-derived compounds with CT may enhance short-term, outcome-specific efficacy. TG + TOP showed the greatest PASI reduction. Immunologically, CG-based regimens optimized IL-23/IL-17 suppression and CD4^+^/CD8^+^ normalization, while TGP + TOP maximized TNF-*α* inhibition. These distinct profiles inform individualized treatment and warrant long-term confirmation.

**Systematic review registration:**

https://www.crd.york.ac.uk/PROSPERO/view/CRD420261304681, identifier (CRD420261304681).

## 1 Introduction

Psoriasis vulgaris (PV) is a chronic immune-mediated inflammatory skin disease characterized by recurrent erythematous scaly plaques and substantial impairment in quality of life. With a global prevalence of approximately 2–3%, PV imposes considerable psychological, social, and economic burdens on affected individuals (1). The pathogenesis of PV is closely associated with dysregulation of the IL-23/Th17 immune axis, excessive keratinocyte proliferation, and persistent release of inflammatory cytokines, such as IL-17, IL-23, and TNF-*α* (2). Current therapeutic strategies include topical agents, phototherapy, conventional therapy (CT), and biologics. Although these treatments can effectively improve skin lesions, their long-term use is often limited by adverse effects, relapse after discontinuation, infection risk, high costs, and reduced patient adherence (2). Therefore, safer and more effective long-term therapeutic approaches remain an important clinical need.

Plant-derived bioactive compounds have long been used to treat PV. Recent attention has focused on standardized bioactive extracts and active derivatives with well-characterized pharmacological mechanisms, such as compound glycyrrhizin (CG), tripterygium glycosides (TG), and total glucosides of paeony (TGP). These agents target critical PV pathways—suppressing Th17 responses and reducing IL-17, IL-23, and TNF-*α*—thereby modulating immunity and improving skin lesions (3–6). Several randomized controlled trials (RCTs) have shown promise for oral compounds as monotherapy or adjunctive therapy with CT (4, 5). However, significant evidence gaps remain: most systematic reviews evaluate individual compounds against controls but lack head-to-head comparisons. Furthermore, systematic rankings of efficacy and safety across different drug regimens have not been established. Existing analyses focus primarily on clinical scores, such as the psoriasis area and severity index (PASI). Immune biomarkers, such as CD4^+^/CD8^+^ ratios, IL-17, IL-23, and TNF-*α*, have been underexplored, limiting mechanistic insights into treatment-specific responses.

Network meta-analysis synthesizes direct and indirect evidence to simultaneously compare multiple interventions. Therefore, this study aimed to compare and rank the efficacy and safety of plant-derived bioactive compounds and their combination regimens in PV using network meta-analysis. The primary outcome was PASI improvement, while secondary outcomes included inflammatory biomarkers and adverse events.

## 2 Methods

We designed and wrote this paper according to the Preferred Reporting Items for Systematic Reviews and meta-analyses (PRISMA) 2020 statement (7) and registered the protocol with PROSPERO (CRD420261304681).

### 2.1 Search strategy

We searched PubMed, Embase, Cochrane Library, Web of Science, China National Knowledge Infrastructure (CNKI), SinoMed, and Wanfang databases from inception to 5 November 2025 for RCTs, evaluating oral plant-derived bioactive compounds in PV treatment. Compounds of interest included CG, TG, TGP, sinomenine, *Tripterygium hypoglaucum*, Colquhounia root, Zhengqingfengtongning, *Daphne giraldii* Nitsche, and *Aconitum brachypodum* Diels. The complete search strategy for each database, such as keywords, Boolean operators, and database-specific filters, is provided in Supplementary material 1.1.

### 2.2 Eligibility criteria

The inclusion and exclusion criteria were cooperatively established by two researchers (Yue Du and Shouxu Zhang).

#### 2.2.1 Inclusion criteria

We included studies meeting the following criteria: (1) participants diagnosed with PV according to the 2018 Chinese Medical Association guidelines (8) or established diagnostic criteria; (2) intervention groups that received oral plant-derived bioactive compounds (CG, TG, TGP, sinomenine, *Tripterygium hypoglaucum*, Colquhounia Root, Zhengqingfengtongning, *Daphne giraldii* Nitsche, or *Aconitum brachypodum* Diels) as monotherapy or combined with CT (acitretin, calcipotriol, or NB-UVB); (3) control groups received oral compounds, placebo, or CT; and (4) outcomes included at least one of the following: (a) the primary outcome, PASI score; or (b) secondary outcomes, such as the CD4^+^/CD8^+^ ratio, serum IL-23, serum IL-17, or serum TNF-*α*. Adverse events were additionally extracted as safety outcomes when reported, and (5) the study design was an RCT.

#### 2.2.2 Exclusion criteria

We excluded studies meeting any of the following criteria: (1) non-PV subtypes (erythrodermic, pustular, or psoriatic arthritis); (2) publications in languages other than English or Chinese; (3) duplicate publications; (4) studies with unobtainable full text or insufficient data for extraction; (5) non-randomized designs, such as animal experiments, meta-analyses, reviews, conference abstracts, dissertations, letters/correspondence, guidelines, or case reports; (6) non-oral administration routes; and (7) studies combining compounds with commercial Chinese polyherbal preparation or complex herbal formulas.

### 2.3 Study selection and data extraction

Two reviewers (Xiaoxuan Chen and Tian Lv) independently screened the literature and extracted data. All retrieved records were imported into EndNote 22, and duplicates were removed. We developed a standardized Excel spreadsheet to extract the following data: first author, publication year, sample size, mean age, disease duration, intervention type and duration, treatment course, and outcome measures. Reviewers screened titles and abstracts first, then assessed full texts. A third reviewer (Lingling Yuan) resolved disagreements through discussion and consultation of the original publications when needed. When standard deviations were not reported, means were extracted and presented descriptively.

### 2.4 Quality assessment

We assessed risk of bias using the Cochrane Risk of Bias Tool 2.0 (RoB2.0) (9), which evaluates five domains: randomization process, deviations from intended interventions, missing outcome data, measurement of the outcome, and selection of the reported result. Each domain was rated as low risk, some concerns, or high risk based on responses to specific signaling questions within the tool. Two independent reviewers (Yue Du and Guangshan Chen) conducted the assessment, with disagreements resolved by a third reviewer (Lingling Li).

### 2.5 Statistical analysis

We performed network meta-analysis using the mvmeta package in Stata 16.0. For continuous outcomes, mean differences and standard deviations were used as effect measures. Of the four included multi-arm trials, one study with different dose groups was pooled into a single arm, while the remaining three were split into pairwise comparisons. Data pooling formulas are provided in Supplementary material 1.2. Evidence quality and risk of bias were assessed using RevMan 5.3, and 95% confidence intervals (CIs) were reported for all effect sizes. Between-study heterogeneity was evaluated using prediction intervals, and local inconsistency (i.e., statistical differences between direct and indirect results) was assessed using node-splitting methods. Network transitivity (i.e., studies included in different comparisons are sufficiently similar in clinical and methodological characteristics) was evaluated by comparing these characteristics across studies. League tables were used to present pairwise comparisons between interventions. Statistical significance was set at *α* = 0.05. When closed loops existed among interventions, inconsistency tests were performed to evaluate agreement between direct and indirect comparisons. Interventions were ranked using the surface under the cumulative ranking curve (SUCRA), which estimates the probability that each intervention ranks highest based on available evidence. SUCRA reflects relative ranking probabilities rather than absolute clinical superiority. As an exploratory tool, SUCRA should be interpreted alongside effect estimates, study quality, sample sizes, and publication bias, and should not serve as the sole basis for clinical decision-making. Publication bias was assessed using comparison-adjusted funnel plots generated in Stata 16.0.

## 3 Results

### 3.1 Literature search and screening

**Figure 1:**
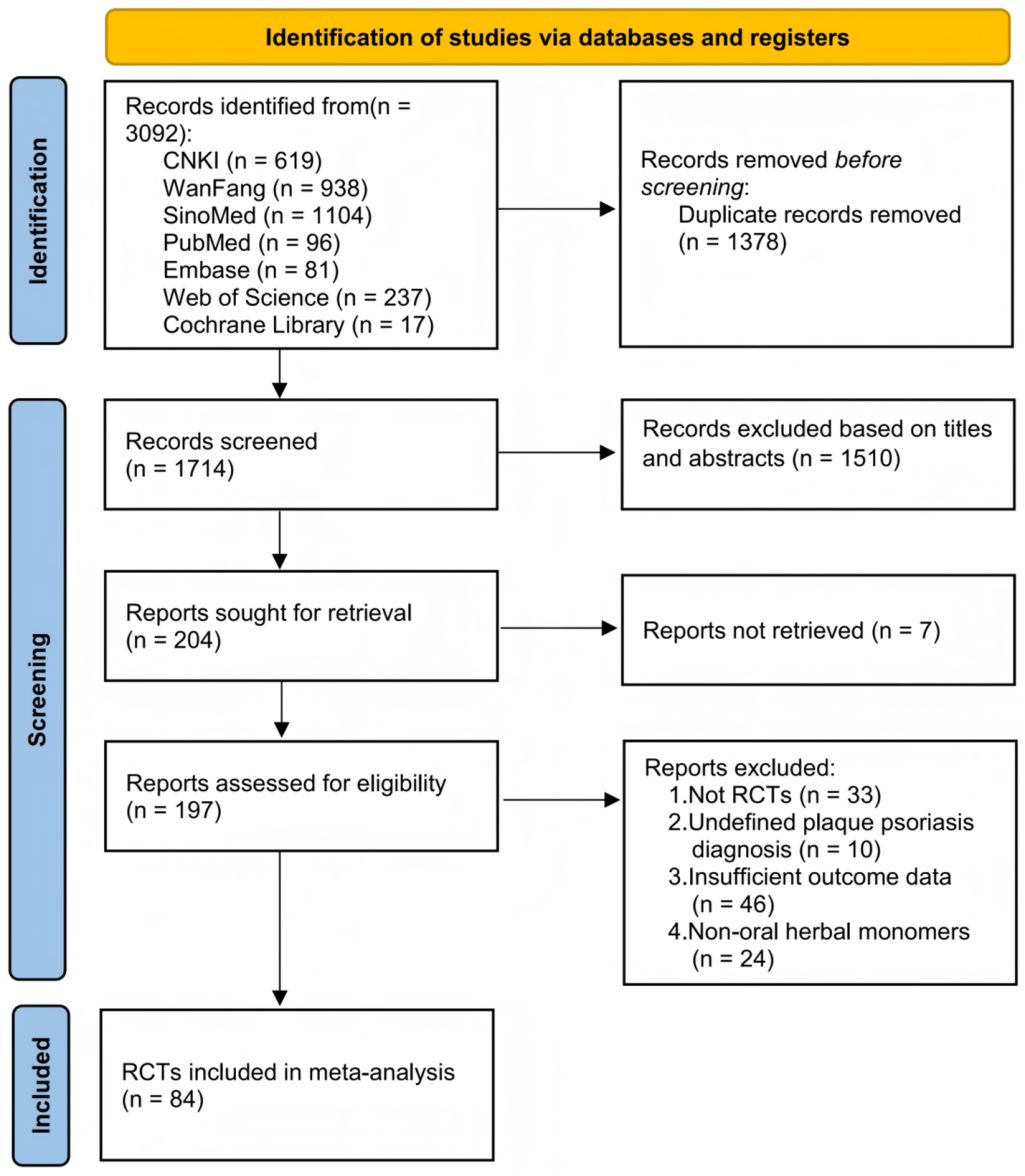
PRISMA flowchart of the literature search and selection. This flow diagram shows the process used to identify relevant records for the network meta-analysis. The systematic literature search was performed from database inception to 5 November 2025; RCT, randomized controlled trial.

The initial search yielded 3,092 records. After removing duplicates, we screened 1,714 titles and abstracts, of which 197 were retrieved for full-text assessment. Ultimately, 84 studies met the inclusion criteria and were included in the meta-analysis (10–93). The step-by-step literature screening and selection process is detailed in Figure 1.

### 3.2 Study characteristics

The final analysis included 84 RCTs enrolling 7,544 participants. All studies were conducted in China across various clinical settings (outpatient and inpatient). Four trials used three-arm designs (47, 50, 91, 93), while the remainder were two-arm parallel trials. Most participants were adults with PV, and treatment duration ranged from 4 to 12 weeks. Although our initial search strategy targeted nine distinct bioactive compounds, only trials investigating CG, TG, and TGP met all predefined inclusion criteria, as the others lacked eligible RCTs. Consequently, the analyzed interventions focused exclusively on these three plant-derived compounds. Across the included trials, all intervention groups received one of these compounds combined with CT, while control groups received CT alone (acitretin, NB-UVB, or topical calcipotriol). Most studies reported PASI scores as the primary outcome. To explore the mechanisms of action, several studies also measured inflammatory biomarkers, such as IL-17, IL-23, TNF-*α*, and CD4^+^/CD8^+^ ratios. Detailed study characteristics are provided in the Supplementary material 2.1.

### 3.3 Risk of bias assessment

**Figure 2:**
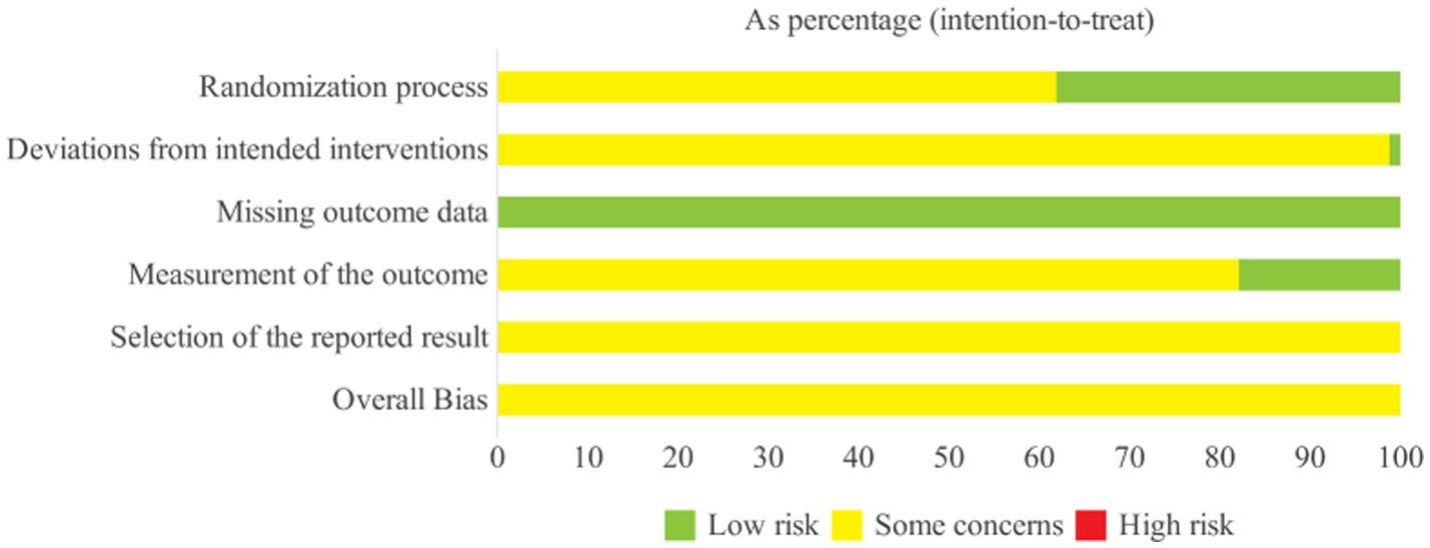
Percent of studies with categories for risk of bias.

All 84 included studies reported comparable baseline characteristics between groups. For randomization, 32 studies (10, 12, 15, 17, 25, 27, 28, 32, 34–37, 39, 42, 43, 45, 46, 48, 50, 52, 53, 60, 61, 64–66, 68, 79, 85–88) described adequate random sequence generation methods (e.g., random number tables) and were rated as low risk, while the remaining 52 studies merely stated “random allocation” without methodological details and were rated as having some concerns. For deviations from intended interventions, one study used a double-blind design and was rated as low risk. The remaining 83 studies were open−label without blinding and were rated as having some concerns. All studies reported complete outcome data or had minimal, balanced missing data across groups, yielding low risk for this domain. For outcome measurement, one study (the double-blind trial) was rated as low risk because outcome assessors were masked to treatment allocation. An additional 14 studies reporting only objective laboratory outcomes (e.g., serum cytokines) were minimally susceptible to measurement bias. However, the remaining 69 studies relied on PASI scores—a semi-subjective measure—without assessor blinding and were rated as having some concerns. For selective reporting, all 84 studies were rated with some concerns due to the absence of pre-registered protocols or statistical analysis plans. Overall, given deficiencies in one or more domains, all 84 studies were judged to have some concerns for the overall risk of bias. Summary results are presented in Figure 2, which shows stacked bar charts visually quantifying the proportion of risk levels. The graphic clearly demonstrates that while domains such as “missing outcome data” are uniformly low risk, the visual dominance of “some concerns” in the measurement and reporting domains drives the overall risk profile, with detailed study-level assessments provided in Supplementary material 3.1.

### 3.4 Network meta-analysis results

#### 3.4.1 Network evidence diagram

**Figure 3:**
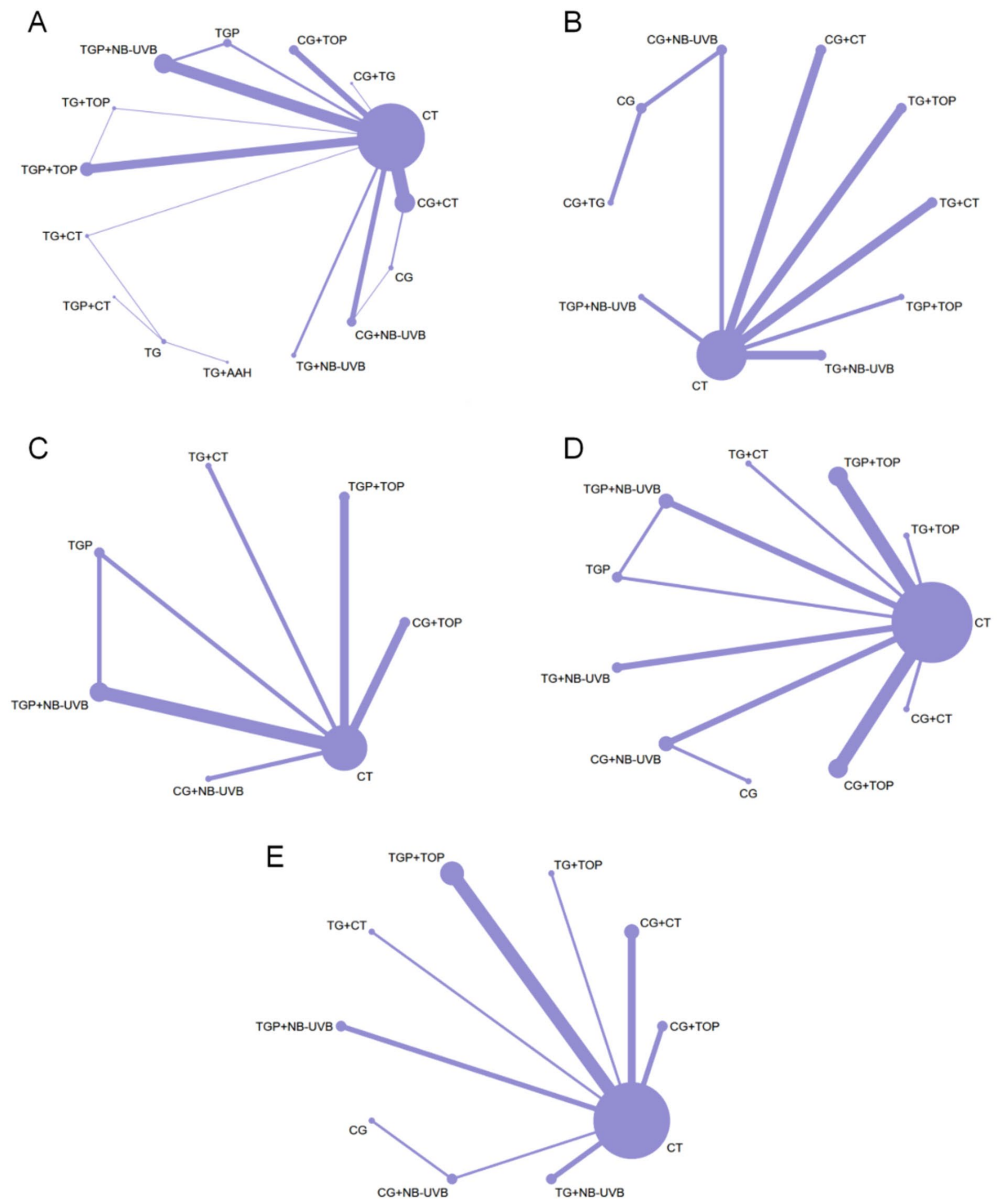
Network plots for PASI **(A)**, CD4^+^/CD8^+^ **(B)**, IL-23 **(C)**, IL-17 **(D)**, and the TNF-α **(E)**. The number of participants in that type of intervention is represented by the size of the nodes, and the number of studies used for that comparison is represented by the thickness of the lines connecting the interventions. CT, conventional therapy; TGP, total glucosides of paeony; TG, tripterygium glycosides; CG, compound glycyrrhizin; NB-UVB, narrow−band ultraviolet B; TOP, topical therapy; AAH, acupoint autohemotherapy.

The 84 studies evaluated 15 interventions. Figure 3 displays the network plots for each outcome. Edge thickness represents the number of direct comparisons between intervention pairs, and node size is proportional to the total sample size for each intervention. In these network plots, the central position and consistently large node size of CT visually emphasize its role as the primary common comparator. Furthermore, the thickest connecting lines radiate between CT and the respective compound combination regimens, indicating that direct head-to-head evidence between different plant compounds is relatively sparse, and the network relies heavily on indirect comparisons.

#### 3.4.2 Psoriasis area and severity index

**Figure 4:**
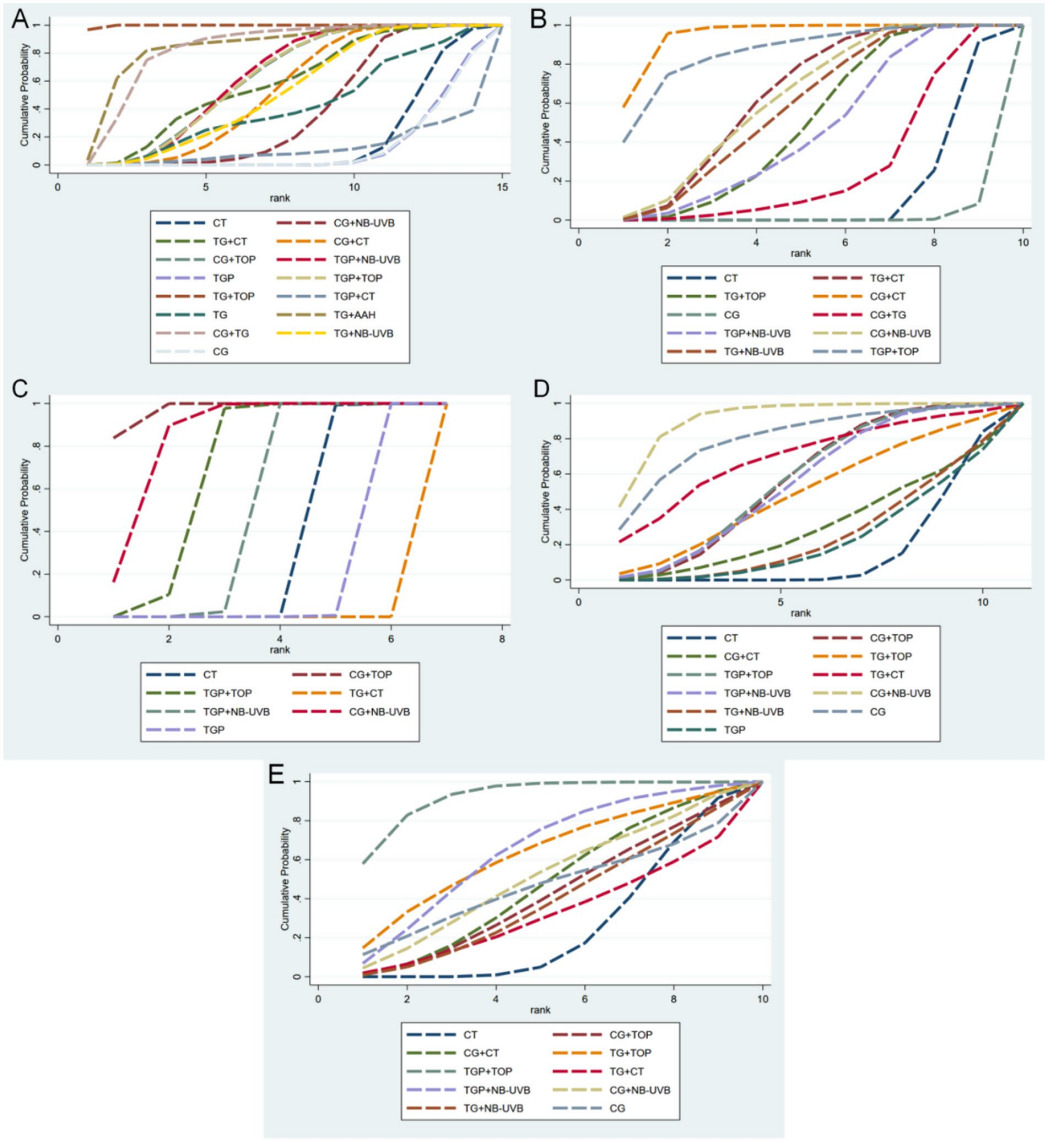
Net meta-analysis ranking results for each outcome indicator. PASI **(A)**, CD4^+^/CD8^+^ **(B)**, IL-23 **(C)**, IL-17 **(D)**, and the TNF-α **(E)**. CT, conventional therapy; TGP, total glucosides of paeony; TG, tripterygium glycosides; CG, compound glycyrrhizin; NB-UVB, narrow-band ultraviolet B; TOP, topical therapy; AAH, acupoint autohemotherapy.

Sixty-four RCTs (10–16, 21–24, 26–29, 31–33, 35, 38–47, 49–58, 60, 63–65, 67, 68, 71–74, 76, 78–84, 86, 87, 89–93) enrolling 5,982 participants reported PASI scores. The network included 15 interventions (TOP denotes topical therapy; AAH denotes acupoint autohemotherapy). Compared with CT alone, nine combination regimens significantly reduced PASI scores: TG + TOP [mean difference (MD) = −13.96, 95% confidence interval (CI) (−16.88, −11.03)], TG + AAH [MD = −7.15, 95% CI (−13.67, −0.63)], CG + TG [MD = −5.74, 95% CI (−8.91, −2.57)], TGP + NB-UVB [MD = −3.42, 95% CI (−4.29, −2.55)], TGP + TOP [MD = −3.39, 95% CI (−4.45, −2.33)], CG + TOP [MD = −3.39, 95% CI (−4.49, −2.30)], CG + CT [MD = −2.98, 95% CI (−3.87, −2.09)], TG + NB-UVB [MD = −2.92, 95% CI (−4.63, −1.21)], and CG + NB-UVB [MD = −2.15, 95% CI (−3.37, −0.93)], and the differences were statistically significant (all *p* < 0.05). Compared with TGP, TG + TOP [MD = −14.45, 95% CI (−17.75, −11.15)], TG + AAH [MD = −7.64, 95% CI (−14.33, −0.94)], CG + TG [MD = −6.23, 95% CI (−9.75, −2.71)], TGP + NB-UVB [MD = −3.91, 95% CI (−5.44, −2.38)], TGP + TOP [MD = −3.88, 95% CI (−5.74, −2.02)], CG + TOP [MD = −3.88, 95% CI (−5.76, −2.00)], CG + CT [MD = −3.47, 95% CI (−5.25, −1.70)], TG + NB-UVB [MD = −3.41, 95% CI (−5.71, −1.12)], and CG + NB-UVB [MD = −2.64, 95% CI (−4.60, −0.68)] all reduced the PASI score (all *p* < 0.05). Compared with CG, TG + TOP [MD = −14.49, 95% CI (−17.96, −11.02)], TG + AAH [MD = −7.68, 95% CI (−14.47, −0.90)], CG + TG [MD = −6.27, 95% CI (−9.96, −2.59)], TGP + NB-UVB [MD = −3.95, 95% CI (−6.03, −1.88)], TGP + TOP [MD = −3.92, 95% CI (−6.09, −1.76)], CG + TOP [MD = −3.93, 95% CI (−6.10, −1.75)], CG + CT [MD = −3.52, 95% CI (−5.31, −1.72)], TG + NB-UVB [MD = −3.46, 95% CI (−6.00, −0.91)], and CG + NB-UVB [MD = −2.68, 95% CI (−4.68, −0.69)], and the differences were statistically significant (all *p* < 0.05). Additionally, TG + TOP [MD = −16.13, 95% CI (−24.14, −8.11)] and TG + AAH [MD = −9.32, 95% CI (−16.34, −2.30)] demonstrated significant advantages over TGP + CT (all *p* < 0.05). Complete pairwise comparisons are detailed in Supplementary material 2.2. The comparative efficacy rankings are shown graphically in the SUCRA curves (Figure 4), where treatments whose curves shift closer to the top-left corner possess a higher probability of being the optimal intervention. Specifically, SUCRA analysis indicated that TG + TOP (99.80%) and TG + AAH (84.60%) ranked highest for PASI score reduction (Figure 4A), as their curves distinctly separate from the others and rise most sharply, with complete rankings for all outcomes presented in Table 1.

**Table 1:** Ranking of the efficacy of each treatment.

Treatment	PASI		CD4^+^/CD8^+^		IL-23		IL-17		TNF-α	
	SUCRA%	Rank	SUCRA%	Rank	SUCRA%	Rank	SUCRA%	Rank	SUCRA%	Rank
CT	17.30%	12	13.10%	9	33.20%	5	15.00%	11	25.00%	10
CG	11.80%	14	1%	10	—	—	80.20%	2	45.80%	6
TG	41.80%	10	—	—	—	—	—	—	—	—
TGP	12.10%	13	—	—	16.80%	6	22.50%	10	—	—
CG + CT	52.90%	8	94.70%	1	—	—	30.60%	8	46.70%	5
TG + CT	58.20%	7	63.70%	3	0	7	68.80%	3	32.10%	9
TGP + CT	11.60%	15	—	—	—	—	—	—	—	—
CG + TOP	61.90%	6	—	—	97.30%	1	56.30%	5	41.40%	7
TG + TOP	99.80%	1	49.60%	6	—	—	49.00%	7	62.90%	3
TGP + TOP	62.00%	5	86.00%	2	68.10%	3	56.70%	4	92.40%	1
CG + NB-UVB	37.90%	11	62.20%	4	84.20%	2	91.20%	1	50.60%	4
TG + NB-UVB	52.00%	9	57.70%	5	—	—	24.90%	9	38.40%	8
TGP + NB-UVB	62.90%	4	45.80%	7	50.40%	4	54.90%	6	64.70%	2
CG + TG	83.40%	3	26.20%	8	—	—	—	—	—	—
TG + AAH	84.60%	2	—	—	—	—	—	—	—	—

—, not mentioned. Data are expressed as mean ± SD. CT, conventional therapy; TGP, total glucosides of paeony; TG, tripterygium glycosides; CG, compound glycyrrhizin; NB-UVB, narrow-band ultraviolet B; TOP, topical therapy; AAH, acupoint autohemotherapy.

#### 3.4.3 CD4^+^/CD8^+^

Thirteen RCTs (34, 38–40, 48, 59, 62, 68, 70, 77, 85–87) enrolling 1,079 participants reported CD4^+^/CD8^+^ ratios. The network included 10 interventions. Compared with CT, CG + CT [MD = 0.58, 95% CI (0.42, 0.74)], TGP + TOP [MD = 0.53, 95% CI (0.20, 0.86)], TG + CT [MD = 0.35, 95% CI (0.23, 0.47)], CG + NB-UVB [MD = 0.34, 95% CI (0.15, 0.53)], TG + NB-UVB [MD = 0.32, 95% CI (0.16, 0.49)], TG + TOP [MD = 0.29, 95% CI (0.16, 0.41)], and TGP + NB-UVB [MD = 0.26, 95% CI (0.05, 0.47)] significantly regulated the CD4^+^/CD8^+^ ratio (all *p* < 0.05). Compared with CG, CG + CT [MD = 0.76, 95% CI (0.46, 1.06)], TGP + TOP [MD = 0.71, 95% CI (0.29, 1.13)], TG + CT [MD = 0.53, 95% CI (0.25, 0.81)], CG + NB-UVB [MD = 0.52, 95% CI (0.35, 0.69)], TG + NB-UVB [MD = 0.50, 95% CI (0.20, 0.81)], TG + TOP [MD = 0.47, 95% CI (0.18, 0.75)], TGP + NB-UVB [MD = 0.44, 95% CI (0.11, 0.77)], and CG + TG [MD = 0.29, 95% CI (0.12, 0.46)] could significantly regulated the CD4^+^/CD8^+^ ratio, and the differences were statistically significant (all *p* < 0.05). CG + CT significantly improved T lymphocyte subset distribution compared with CG + TG [MD = 0.47, 95% CI (0.12, 0.82)], TGP + NB-UVB [MD = 0.32, 95% CI (0.06, 0.58)], TG + TOP [MD = 0.29, 95% CI (0.09, 0.50)], TG + NB-UVB [MD = 0.26, 95% CI (0.03, 0.49)], and TG + CT [MD = 0.23, 95% CI (0.03, 0.43)] (all *p* < 0.05). Other pairwise comparisons showed no significant differences (Supplementary material 2.3). SUCRA analysis indicated that CG + CT (94.70%) and TGP + TOP (86.00%) ranked highest for improving CD4^+^/CD8^+^ ratios (Figure 4B).

#### 3.4.4 Il-23

Nine RCTs (21, 25, 26, 30, 50, 55, 66, 69, 70) enrolling 889 participants reported serum IL-23 levels. The network included seven interventions. Network meta-analysis demonstrated significant reductions in IL-23 for all pairwise comparisons except CG + TOP versus CG + NB-UVB and CG + NB-UVB versus TGP + TOP, which showed no significant differences (all *p* > 0.05). All other pairwise comparisons were statistically significant (all *p* < 0.05). Detailed effect estimates are provided in Supplementary material 2.4. SUCRA analysis indicated that CG + TOP (97.30%) and CG + NB-UVB (84.20%) ranked highest for reducing IL-23 (Figure 4C).

#### 3.4.5 Il-17

Eighteen RCTs (18–21, 25, 30, 34, 36, 37, 39, 45, 50, 55, 61, 66, 69, 70, 86) enrolling 1,816 participants reported serum IL-17 levels. The network included 11 interventions. Compared with CT alone, CG + NB-UVB [MD = −29.99, 95% CI (−46.25, −13.74)], TGP + TOP [MD = −13.05, 95% CI (−24.62, −1.48)], and CG + TOP [MD = −12.88, 95% CI (−23.30, −2.47)] significantly reduced IL-17 levels (all *p* < 0.05). CG + NB-UVB also demonstrated significant superiority over TGP [MD = −28.87, 95% CI (−52.04, −5.71)] and TG + NB-UVB [MD = −27.77, 95% CI (−49.60, −5.93)] (*p* < 0.05). Other pairwise comparisons showed no significant differences (Supplementary material 2.5). SUCRA analysis indicated CG + NB-UVB (91.20%) and CG monotherapy (80.20%) ranked highest for reducing IL-17 (Figure 4D).

#### 3.4.6 TNF-*α*

Eighteen RCTs (17, 19, 21, 26, 35, 36, 45, 49, 53, 55, 56, 60, 61, 66, 75, 83, 86, 88) enrolling 1,836 participants reported serum TNF-α levels. The network included 10 interventions. Compared with CT alone, TGP + TOP [MD = −18.54, 95% CI (−27.74, −9.35)] significantly reduced TNF-*α* levels (*p* < 0.05). TGP + TOP also demonstrated significant superiority over TG + NB-UVB [MD = −16.18, 95% CI (−31.83, −0.52)] and CG + CT [MD = −14.14, 95% CI (−28.00, −0.28)] (*p* < 0.05). There were no statistically significant differences in other comparisons (all *p* > 0.05). Other pairwise comparisons showed no significant differences (Supplementary material 2.6). SUCRA analysis indicated TGP + TOP (92.40%) and TGP + NB-UVB (64.70%) ranked highest for reducing TNF-α (Figure 4E).

#### 3.4.7 Adverse reactions

Adverse event (AE) data were reported in 63 included RCTs. Most reported AEs were mild to moderate and improved after symptomatic treatment, dose adjustment, or treatment discontinuation. No treatment-related deaths or irreversible serious adverse events were reported. Detailed AEs information is summarized in Supplementary material 2.7.

Gastrointestinal symptoms were among the most commonly reported AEs in TG- and TGP-containing regimens, such as diarrhea, nausea, abdominal discomfort, vomiting, and loss of appetite. Mild elevations in hepatic transaminases or abnormal liver function were occasionally reported in both TG- and TGP-containing regimens. Regimens containing acitretin or NB-UVB were more frequently associated with mucocutaneous reactions, such as dry skin, cheilitis, dry eyes, erythema, pruritus, desquamation, and burning sensations. Laboratory abnormalities, such as hyperlipidemia and elevated hepatic transaminases, were also reported, mainly in acitretin-containing regimens. CG-containing regimens were primarily associated with mild localized skin irritation or transient skin discomfort.

Other less frequently reported AEs included dizziness, headache, fatigue, leukopenia, menstrual irregularities, abnormal blood counts, and influenza-like symptoms. Overall, the included plant-derived bioactive compound regimens demonstrated generally acceptable tolerability in patients with PV.

#### 3.4.8 Assessment of publication bias

**Figure 5:**
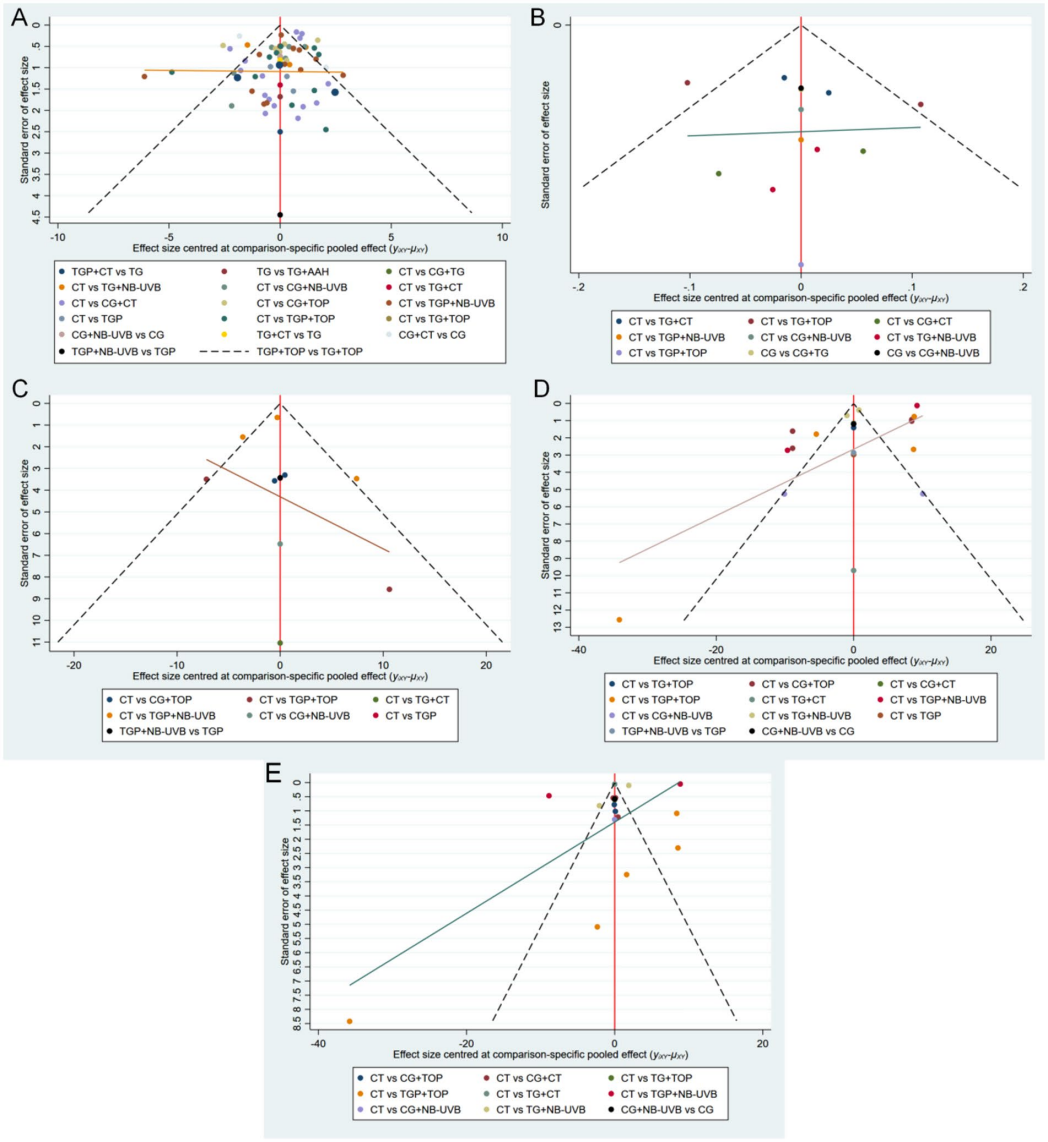
Comparison−correction funnel plot of each outcome indicator. PASI **(A)**, CD4^+^/CD8^+^ **(B)**, IL-23 **(C)**, IL-17 **(D)**, and TNF-α **(E)**. CT, conventional therapy; TGP, total glucosides of paeony; TG, Tripterygium glycosides; CG, compound glycyrrhizin; NB-UVB, Narrow-band ultraviolet B; TOP, topical therapy; AAH, acupoint autohemotherapy.

We generated comparison-adjusted funnel plots for all 84 studies to visually assess small-study effects, where the y-axis represents standard error (precision) and the x-axis represents the effect size (Figure 5). For PASI scores and CD4^+^/CD8^+^ ratios, data points were distributed symmetrically around the regression line and concentrated near the funnel apex, with near-horizontal regression lines, indicating no evidence of publication bias. In contrast, funnel plots for IL-23, IL-17, and TNF-*α* showed asymmetric distributions with scattered data points and inclined regression lines, suggesting potential small-study effects, which are visually characterized by the wider scattering of points in the lower portion of the funnel (trials with larger standard errors).

## 4 Discussion

### 4.1 Summary of the main results

Despite advances in retinoids and biologics for PV management, long-term use remains constrained by hepatotoxicity, high relapse rates, and substantial costs. We conducted a network meta-analysis of 84 RCTs to evaluate the efficacy and safety of three plant-derived bioactive compounds (TG, TGP, and CG) and their various combination regimens. A comparative evaluation suggests that, while standard systemic and topical monotherapies provide foundational efficacy, plant-derived compounds used alone may not consistently outperform them. Distinct clinical advantages emerge primarily when these compounds are combined with conventional modalities, as combination regimens frequently ranked highest in SUCRA for multiple clinical and immunological endpoints, indicating potential benefit as adjunctive interventions.

To translate these statistical rankings into clinical practice, a stratified approach based on patient profiles may be considered. For patients presenting with high disease severity where rapid lesion clearance is the primary clinical objective, the TG + TOP regimen, which demonstrated the highest probability of PASI reduction (SUCRA = 99.80%), could be prioritized as a short-term induction strategy. However, given its potential gastrointestinal and hepatic risks, it may not be optimal for indefinite maintenance. Conversely, for patients requiring long-term disease control with prominent immunologic activity, CG-based combinations appear to offer a promising alternative. Specifically, CG + CT demonstrated the highest probability of restoring the CD4^+^/CD8^+^ ratio balance (SUCRA = 94.70%). Furthermore, CG-containing regimens were associated with an enhanced potential in suppressing the IL-23/IL-17 axis. Notably, CG + TOP showed the highest probability for IL-23 suppression (SUCRA = 97.30%), and CG + NB-UVB may act as a comprehensive axis modulator, ranking first in reducing IL-17 (SUCRA = 91.20%) and second in IL-23 suppression (SUCRA = 84.20%). Additionally, TGP + TOP ranked highest for TNF-*α* suppression (SUCRA = 92.40%) and second for CD4^+^/CD8^+^ ratio normalization (SUCRA = 86.00%). These differential rankings across inflammatory pathways highlight opportunities for individualized treatment selection, balancing efficacy and safety.

### 4.2 Comparison with other studies

Prior systematic reviews and meta-analyses have supported the general therapeutic benefits of individual agents such as TGP or TG when combined with CT (4, 5). However, they typically evaluated these compounds in isolation against standard controls, leaving their comparative efficacy poorly defined. The present study extends existing evidence by leveraging both direct and indirect evidence to simultaneously compare and rank 15 distinct treatment regimens, thereby addressing a critical evidence gap in head-to-head clinical trials. Crucially, unlike existing analyses that predominantly focus on subjective clinical endpoints such as the PASI score, this study systematically integrates objective immunological biomarkers, such as peripheral CD4^+^/CD8^+^ ratios and serum cytokines (IL-17, IL-23, TNF-*α*). This dual approach not only provides a mechanism-aligned framework for evaluating treatment responses but also partially mitigates the risk of observer bias inherent in the open-label designs of the included primary trials. Furthermore, from a clinical safety perspective, while conventional therapies (such as acitretin monotherapy) are frequently constrained by dose-limiting toxicities, the integration of these plant-derived bioactive compounds suggests a potential “dose-sparing” benefit. This may allow clinicians to optimize efficacy while minimizing exposure to more toxic conventional agents, offering a cost-effective and systemically tolerable alternative for patients who may not be candidates for biologic therapies.

### 4.3 Mechanism analysis

PV is a chronic inflammatory condition maintained by an imbalance between innate and adaptive immunity. Its core pathological feature involves overactivation of the IL-23/Th17 axis, keratinocyte hyperproliferation, and a positive feedback cycle among the inflammation-immune-barrier axis. Therefore, interventions that can modulate multiple key pathological nodes and immune pathways may have true potential for disease regulation. The compounds included in this study (CG, TG, and TGP) showed differentiated efficacy rankings in the network meta-analysis, which may reflect differences in their molecular targets and depth of immune pathway modulation.

CG, a derivative of glycyrrhizic acid (the primary active component of licorice), exhibits a significant advantage in regulating the IL-23/IL-17 axis, which is closely related to its precise intervention in downstream key signaling pathways. CG has been confirmed to be an effective agonist of SIRT1, which can upregulate the expression of SIRT1 and thereby inhibit STAT3, phosphorylated STAT3 (p-STAT3), and acetylated STAT3 (a-STAT3). This not only reduces its transcriptional activity but also enhances the negative feedback regulation of proinflammatory signals by cells (3). This mechanism plays a key role in the regulation of the terminal differentiation of KCs, which helps to slow down the excessive proliferation and inflammatory response of KCs and improve skin lesions. Meanwhile, CG has a significant negative regulatory effect on the NF-κB/MAPK pathway, which can downregulate the expression of the endothelial cell surface adhesion molecule ICAM-1, thereby reducing the expression of proinflammatory genes and inflammatory cell infiltration (94). Preclinical studies report hepatoprotective effects of glycyrrhizin, which may be relevant in mitigating hepatotoxicity associated with concomitant systemic therapies. These pharmacological properties may contribute to the clinical biomarker effects observed in CG-containing regimens.

In contrast, TG, a standardized active extract rich in diterpenoids with strong immunosuppressive effects derived from the *Tripterygium wilfordii*, has a solid molecular biological basis in the rapid removal of skin lesions and improvement of PASI scores. The continuous overactivation of the IL-23/Th17 axis serves as the primary driver for PV pathogenesis. TG and its main active ingredients exert their effects by highly targeting this pathway. Studies have confirmed that TG can precisely downregulate the phosphorylation level of STAT3. This effect is of crucial significance because STAT3 not only controls the differentiation of Th17 cells but also determines their ability to secrete pathogenic cytokines such as IL-17A, IL-17F, and IL-22 (95). More in-depth research has found that the role of TG is not limited to systemic immunity. It can also significantly reduce the recruitment of γδT17 cells in skin lesions and draining lymph nodes, and improve the local immune-inflammatory microenvironment by reducing the expression of cytokines such as IL-17A, IL-23, and TNF-*α* (96). This regulation of the abundance of local immune cells in the skin, combined with its inhibition of the NF-κB pathway in KCs, effectively curbs the proinflammatory chemotactic feedback loop mediated by CXCL1 and IL-36α (96, 97). This synergistic effect in systemic immunity and local skin inflammation is precisely the pharmacological basis for TG to rapidly control the severity of skin lesions in clinical practice.

In contrast, TGP, a mixture of glycosides extracted from the dried roots of *Paeonia lactiflora*, exhibits multi-target immunomodulatory effects. At the molecular level, TGP can inhibit keratinocyte proliferation and improve pathological changes in psoriasis lesions by suppressing STAT1/STAT3 phosphorylation and reducing inflammatory molecules, such as IL-17A, IL-22, and RORγt (98). It also improves the imbalanced Th17/Treg ratio and promotes the adjustment of immune homeostasis by regulating the immunomodulatory characteristics of skin mesenchymal stem cells (99). TGP has been confirmed to affect multiple key pathways in autoimmune and inflammatory studies. For instance, JAK/STAT, NF-κB, MAPK, and PI3K/Akt/mTOR are all involved in the activation of inflammatory cells, cytokine production, and cell proliferation. Through the coordinated regulation of these pathways, TGP can comprehensively alleviate chronic inflammatory responses (100). In addition, for the characteristic dermal papillary layer vascular dilation and exudation of PV, TGP has demonstrated clear antiangiogenic activity, which can inhibit abnormal angiogenesis and improve local microcirculation by reducing the vascular endothelial growth factor (VEGF) level at the lesion site (101). These mechanisms may contribute to the biomarker improvements observed in TGP-containing regimens.

**Figure 6:**
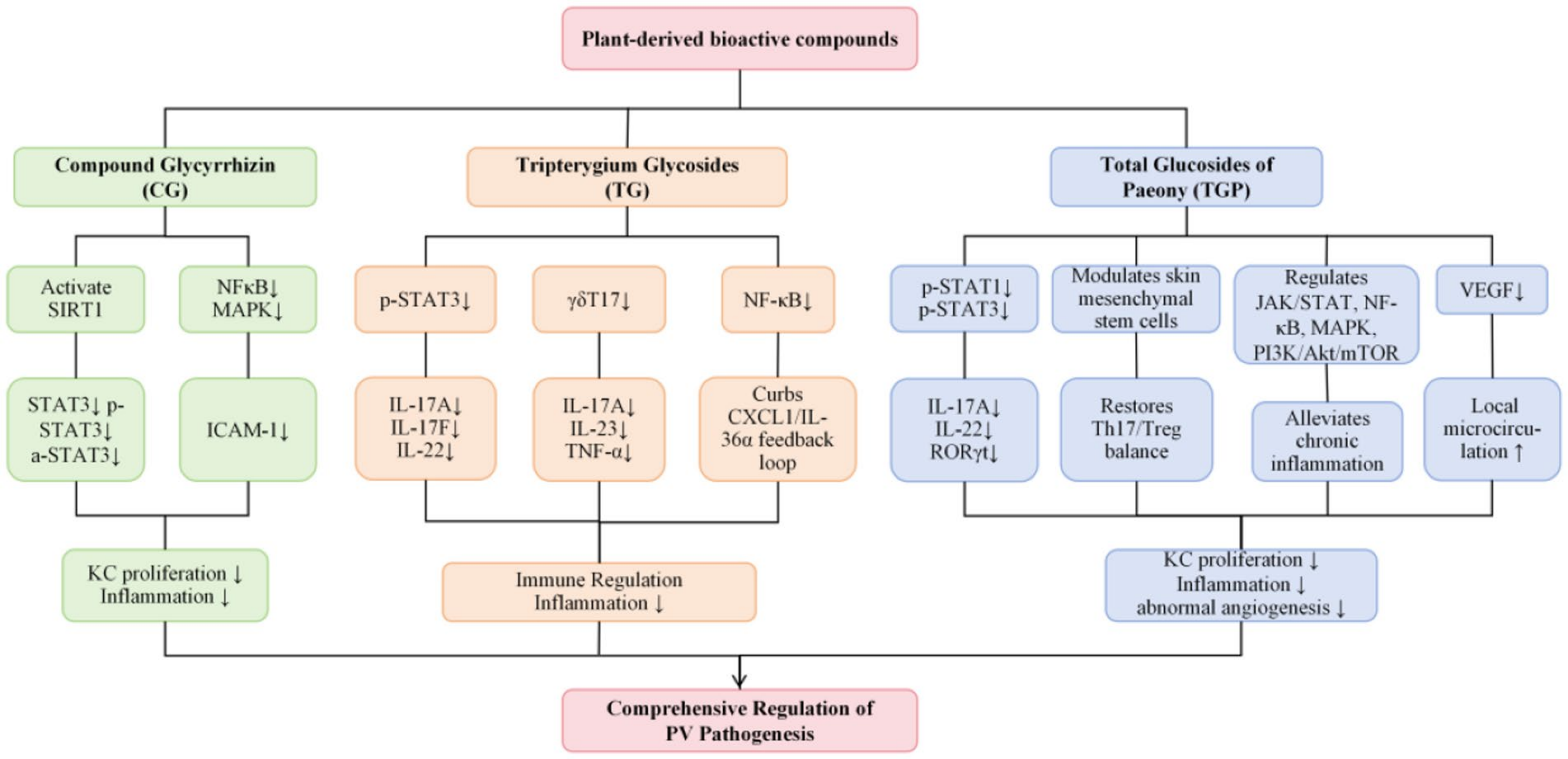
Mechanistic pathways of plant-derived bioactive compounds (CG, TG, and TGP) in the comprehensive regulation of psoriasis vulgaris (PV). This flowchart illustrates the distinct but complementary pharmacological targets of CG, TG, and TGP. CG reduces keratinocyte (KC) proliferation and inflammation by activating SIRT1 to suppress STAT3 (p-STAT3/a-STAT3) and inhibiting NF-κB/MAPK to downregulate ICAM-1. TG regulates immunity by downregulating p-STAT3 (reducing IL-17A/F, IL-22), inhibiting γδT17 cells, and blocking the NF-κB-mediated CXCL1/IL-36α feedback loop. TGP suppresses KC proliferation, inflammation, and abnormal angiogenesis by decreasing p-STAT1/3, modulating skin mesenchymal stem cells to restore Th17/Treg balance, regulating multiple pathways (JAK/STAT, NF-κB, MAPK, PI3K/Akt/mTOR), and suppressing VEGF to improve microcirculation. Together, these parallel interventions provide a comprehensive regulation of PV pathogenesis. (↓): inhibition/down-regulation; (↑): promotion/improvement. PV, psoriasis vulgaris; SIRT1, Sirtuin 1; STAT3, signal transducer and activator of transcription 3; p-STAT, phosphorylated STAT; a-STAT, acetylated STAT; NF-κB, nuclear factor kappa B; MAPK, mitogen-activated protein kinase; ICAM-1, intercellular adhesion molecule 1; KC, keratinocyte; IL, interleukin; TNF-α, tumor necrosis factor alpha; CXCL1, chemokine (C-X-C motif) ligand 1; RORγt, RAR-related orphan receptor gamma; Treg, regulatory T cells; JAK, Janus kinase; PI3K, phosphoinositide 3-kinase; Akt, protein kinase B; mTOR, mammalian target of rapamycin; VEGF, vascular endothelial growth factor.

Figure 6 summarizes the key mechanisms of action of the three plant-derived compounds. In conclusion, the differential performance of individual compounds may underlie observed clinical and biomarker differences and suggest distinct but complementary mechanisms of action. Future studies using single-cell sequencing or proteomics could elucidate how these compounds interact with specific immune cell subsets, thereby supporting more rational combination strategies and personalized treatment selection.

### 4.4 Safety considerations

Safety and tolerability are critical in psoriasis management, particularly for long-term care. Across the 63 RCTs reporting safety data, the investigated regimens were generally well-tolerated with no serious AEs, yet their safety profiles varied distinctly. Conventional acitretin frequently caused mucocutaneous reactions (e.g., severe dryness, cheilitis) and hyperlipidemia. In contrast, TG- and TGP-containing regimens were primarily associated with gastrointestinal symptoms and occasional mild transaminase elevations, while CG-based therapies exhibited a highly favorable systemic safety profile, mostly causing mild local irritation.

Therefore, SUCRA efficacy rankings must be balanced against these specific safety profiles rather than relying solely on PASI scores. Although TG + TOP showed the highest probability of PASI reduction (SUCRA 99.80%), its gastrointestinal and hepatic risks may limit its suitability for indefinite maintenance, especially in patients with baseline organ vulnerabilities. Similarly, while TGP avoids retinoid-induced lipid abnormalities, its potential for mild hepatic and gastrointestinal events necessitates appropriate monitoring. Conversely, CG-based combinations represent a more systemically tolerable alternative for extended immunomodulation. Nevertheless, the relatively short follow-up periods (4–12 weeks) and variable AEs reporting standards across the included trials limit the evaluation of long-term safety. Further large-scale, high-quality RCTs with extended follow-up are required to fully elucidate the long-term tolerability of these integrative regimens.

### 4.5 Limitations and strengths

This network meta-analysis has several strengths. First, we systematically compared multiple combination regimens of plant-derived bioactive compounds, providing a relative ranking of efficacy using SUCRA values. Second, the study additionally incorporated both clinical outcomes (PASI scores) and key inflammatory biomarkers (IL-17, IL-23, TNF-*α*, and CD4^+^/CD8^+^ ratio), offering insight into differential treatment effects. Third, the analysis included 84 RCTs enrolling 7,544 participants, providing substantial statistical power. Finally, our study provides critical comparative evidence against conventional systemic therapies, identifying potential plant-based candidates that may offer better tolerability profiles, thus offering a practical evidence base for clinical decision-making.

This study also has several limitations that should be considered when interpreting the findings. First, the follow-up duration of most included RCTs was relatively short (generally 4–12 weeks), limiting the ability to assess long-term efficacy and safety. Second, AEs reporting varied across studies in terms of definition, severity grading, and completeness, which may have affected the comparability of safety profiles. Third, head-to-head trials directly comparing different plant-derived bioactive compounds or their combination regimens with CT were limited. As a result, some comparative conclusions rely on indirect evidence within the network meta-analysis, which may introduce uncertainty. Fourth, heterogeneity in patient populations, intervention doses, and concomitant therapies across studies may have influenced effect estimates. Finally, grey literature and unpublished studies were not systematically searched, which may contribute to potential publication bias.

## 5 Conclusion

This network meta-analysis of 84 RCTs suggests that oral plant-derived bioactive compounds combined with CT may significantly improve short-term clinical outcomes and immunological parameters in PV. TG + TOP ranked highest for PASI score reduction, indicating potential efficacy for lesion clearance. CG-based regimens emerged as promising options for IL-23/IL-17 suppression and CD4^+^/CD8^+^ ratio normalization, while TGP + TOP demonstrated the most favorable results in TNF-*α* inhibition. Although these findings propose a theoretical framework for individualized integrated treatment strategies based on specific immunophenotypes, the current methodological limitations and short follow-up periods necessitate cautious clinical application. Further high-quality, long-term studies are required to definitively establish these integrative regimens in standard practice.

## Data Availability

The original contributions presented in the study are included in the article/Supplementary material, further inquiries can be directed to the corresponding author/s.
