# Supplementary material for: Efficacy and safety of plant-derived bioactive compounds in the treatment of psoriasis vulgaris: a systematic review and network meta-analysis
Source: Front Med (Lausanne). 2026 Jun 22;13:1844822. doi: 10.3389/fmed.2026.1844822 (PMC13333435; doi:10.3389/fmed.2026.1844822)
Supplement: Supplementary file 1 [file Data_Sheet_1.docx]

Supplementary Material

# Supplementary Data

## Search Strategy

- 1. CNKI

TKA=('银屑病'+'白疕'+'牛皮癣') AND TKA=('甘草酸苷'+'雷公藤多苷'+'白芍总苷'+'青藤碱'+'昆明山海棠'+'火把花根'+'正清风痛宁'+'祖师麻'+'雪上一枝蒿'+'中药单体'+'单体化合物') AND FT='随机'

- 1. WanFang Data

主题:("银屑病"or"白疕"or"牛皮癣") and 主题:("甘草酸苷"or"雷公藤多苷"or"白芍总苷"or"青藤碱"or"昆明山海棠"or"火把花根"or"正清风痛宁"or"祖师麻"or"雪上一枝蒿"or"中药单体"or"单体化合物") 全部:(随机)

- 1. SinoMed

"随机"[全部字段:智能]) AND (((((("银屑病"[不加权:扩展])) OR (("银屑病"[常用字段:智能] OR "牛皮癣"[常用字段:智能] OR "白疕"[常用字段:智能])))) AND (("甘草酸苷"[常用字段:智能] OR "雷公藤多苷"[常用字段:智能] OR "白芍总苷"[常用字段:智能] OR "青藤碱"[常用字段:智能] OR "昆明山海棠"[常用字段:智能] OR "正清风痛宁"[常用字段:智能] OR "雪上一枝蒿"[常用字段:智能] OR "火把花根"[常用字段:智能] OR "祖师麻"[常用字段:智能] OR "中药单体"[常用字段:智能] OR "单体化合物"[常用字段:智能]))))

- 1. PubMed

(((((((((((Glycyrrhizin) OR (tripterygium glycosides)) OR (Total glucosides of paeony)) OR (sinomenine)) OR (Tripterygium hypoglaucum)) OR (Colquhounia Root)) OR (Zhengqingfengtongning)) OR (Daphne giraldii Nitsche)) OR (Aconitum Brachypodum Diels)) OR (Chinese herbal monomer)) OR (monomer compound)) AND ((psoriasis [MeSH] OR psoria* [tiab]))

- 1. Web of science

| Search number | Query |
| --- | --- |
| #1 | TS=(psoriasis OR psoria*) |
| #2 | TS=(Glycyrrhizin OR tripterygium glycosides OR Total glucosides of paeony OR sinomenine OR Tripterygium hypoglaucum OR Colquhounia Root OR Zhengqingfengtongning OR Daphne giraldii Nitsche OR Aconitum Brachypodum Diels OR Chinese herbal monomer OR monomer compound) |
| #3 | #1 OR #2 |

- 1. cochrane library

| Search number | Query |
| --- | --- |
| #1 | MeSH descriptor: [Psoriasis] explode all trees |
| #2 | (psoriasis):ti,ab,kw OR (psoria*):ti,ab,kw |
| #3 | #1 OR #2 |
| #4 | (Glycyrrhizin):ti,ab,kw OR (tripterygium glycosides):ti,ab,kw OR (Total glucosides of paeony):ti,ab,kw OR (sinomenine):ti,ab,kw OR (Tripterygium hypoglaucum):ti,ab,kw |
| #5 | (Colquhounia Root):ti,ab,kw OR (Zhengqingfengtongning):ti,ab,kw OR (Daphne giraldii Nitsche):ti,ab,kw OR (Aconitum Brachypodum Diels):ti,ab,kw OR (Chinese herbal monomer):ti,ab,kw |
| #6 | (monomer compound):ti,ab,kw |
| #7 | #4 OR #5 OR #6 |
| #8 | #3 And #7 |

- 1. Embace

| Search number | Query |
| --- | --- |
| #5 | #3 AND #4 |
| #4 | #1 OR #2 |
| #3 | 'glycyrrhizin' OR 'tripterygium glycosides' OR 'total glucosides of paeony' OR 'sinomenine' OR 'tripterygium hypoglaucum' OR 'colquhounia root' OR 'zhengqingfengtongning' OR 'daphne giraldii nitsche' OR 'aconitum brachypodum diels' OR 'chinese herbal monomer' OR 'monomer compound' |
| #2 | 'psoria*' |
| #1 | 'psoriasis'/exp OR psoriasis |

## Formulae for Data Combination

For three-arm trials, the two intervention groups were pooled using the following calculations: For Group A (sample size=N_1_, mean=M_1_, SD=SD_1_) and Group B (sample size=N_2_, mean=M_2_, SD=SD_2_), the combined sample size was calculated as N=N_1_+N_2_, with weighted mean M=(N_1_M_1_+N_2_M_2_)/(N_1_+N_2_). The pooled standard deviation was derived using the following formula:

$\text{SD}\text{=}\sqrt{\frac{\text{(}\text{N}_{\text{1}}\text{-}\text{1)}\text{S}\text{D}_{\text{1}}^{\text{2}}\text{+(}\text{N}_{\text{2}}\text{-}\text{1)}\text{S}\text{D}_{\text{2}}^{\text{2}}\text{+}\frac{\text{N}_{\text{1}}\text{N}_{\text{2}}}{\text{N}_{\text{1}}\text{+}\text{N}_{\text{2}}}\text{(}\text{M}_{\text{1}}^{\text{2}}\text{+}\text{M}_{\text{2}}^{\text{2}}\text{-}\text{2}\text{M}_{\text{1}}\text{M}_{\text{2}}\text{)}}{\text{N}_{\text{1}}\text{+}\text{N}_{\text{2}}\text{-}\text{1}}}$

# Supplementary Tables

## Characteristics of the included studies

| **Study ID** | **Country; setting** | **Blinding;number of arms** | **Sample size（n）** | | **Age(years)** | | **Disease course (years)** | | **Intervention characteristics** | | **Interven-tion time** | **Out- comes** |
| --- | --- | --- | --- | --- | --- | --- | --- | --- | --- | --- | --- | --- |
|  |  |  | **T** | **C** | **T** | **C** | **T** | **C** | **T** | **C** |  |  |
| Wang and Wang(2024) | China;NS | NS;2 | 35 | 35 | 53.16±3.26 | 54.16±3.82 | 5.14±1.56 | 5.44±1.92 | TG+NB-UVB | NB-UVB | 8 | ⑤⑥ |
| Ma and Zhu(2024) | China;NS | NS;2 | 41 | 41 | 42.48±8.13 | 41.53±7.38 | 4.59±1.25 | 4.67±1.06 | TG+NB-UVB | NB-UVB | 12 | ①⑤⑥ |
| Zhang(2022) | China;NS | NS;2 | 56 | 56 | 41.63±2.13 | 41.59±2.11 | 3.63±0.57 | 3.68±0.60 | CG+NB-UVB | CG | 4 | ①②⑤⑥ |
| Wu(2022) | China;NS | NS;2 | 33 | 30 | 66.42±5.05 | 66.83±5.51 | NS | NS | TGP+TOP | TOP | 8 | ②③⑤ |
| Wang(2022) | China;NS | NS;2 | 64 | 64 | 42.68±2.59 | 41.58±2.35 | 5.32±0.74 | 4.68±0.57 | CG+Acitretin | Acitretin | 8 | ① |
| Liu(2022) | China;NS | NS;2 | 30 | 30 | 38.72±1.11 | 38.81±1.20 | 4.23±0.29 | 4.21±0.32 | CG+Acitretin | Acitretin | 8 | ①②⑤ |
| Lin(2022) | China;hospital outpatients | NS;2 | 47 | 47 | 49.07±4.24 | 48.62±4.18 | 4.23±2.14 | 4.16±2.05 | TGP+Acitretin  +Dithranol | Acitretin+  Dithranol | 8 | ④⑤ |
| Wei(2022) | China;NS | NS;2 | 48 | 48 | 38.59±4.36 | 38.68±4.47 | 4.12±1.28 | 4.29±1.36 | CG+NB-UVB | NB-UVB | 8 | ③⑤⑥ |
| Zhang(2021) | China;NS | NS;2 | 52 | 51 | 37.50±2.90 | 37.80±3.10 | 4.30±1.20 | 4.20±1.20 | CG+Acitretin | Acitretin | 8.6 | ① |
| Wang et al.(2021) | China;NS | NS;2 | 30 | 30 | 45.28±9.38 | 47.13±10.27 | 0.29±0.09 | 0.32±0.09 | TG+NB-UVB | NB-UVB | 4 | ② |
| Wang and Li(2021) | China;NS | NS;2 | 43 | 43 | 36.23±2.55 | 36.52±2.28 | 4.33±2.11 | 4.26±2.04 | CG+Fluticasone propionate | Fluticasone propionate | 4 | ①④⑥ |
| Ren and Zhao(2021) | China;NS | NS;3 | 30 | | 43.38±9.67 | | NS | | TGP+NB-UVB | | 12 | ①③⑤ |
|  |  |  | 30 | |  |  | NS | | TGP | |  |  |
|  |  |  | 30 | |  |  | NS | | NB-UVB | |  |  |
| Peng(2021) | China;NS | NS;2 | 42 | 42 | 38.78±4.15 | 38.75±4.13 | 5.41±1.18 | 5.37±1.16 | TG+Acitretin+Thalidomide | Acitretin+Thalidomide | 8 | ② |
| Ma et al.(2021) | China;NS | double-blind;2 | 39 | 39 | 36.32±9.81 | 47.84±8.63 | NS | NS | CG+NB-UVB  +Halometasone  +Calcipotriol | NB-UVB+  Halometasone+  Calcipotriol | 4 | ① |
| Dong(2021) | China;NS | NS;2 | 33 | 33 | 44.31±1.60 | 43.23±1.44 | 5.98±1.89 | 6.31±1.88 | CG+Acitretin | Acitretin | 8 | ⑥ |
| Zhai et al.(2021) | China;hospital out/inpatients | NS;2 | 40 | 40 | 33.02±5.25 | 32.17±5.41 | 0.41±0.13 | 0.39±0.13 | TGP+Acitretin  +Budesonide | Acitretin+  Budesonide | 4 | ① |
| Zhuo et al.(2020) | China;NS | NS;2 | 50 | 50 | 44.10±8.36 | 43.77±8.27 | NS | NS | CG+Tacrolimus  +Ebastine | CG+Tacrolimus | 8 | ① |
| Zhang and Song(2020) | China;NS | NS;2 | 40 | 40 | 42.95±3.48 | 42.95±3.48 | 1.65±0.24 | 1.65±0.24 | CG+Acitretin | CG | 6 | ① |
| Shang et al.(2020) | China;NS | NS;2 | 30 | 30 | 34.6±4.2 | 35.7±5.3 | 5.37±0.23 | 5.21±0.18 | TGP+Desonide | Desonide | 8 | ① |
| Ren et al.(2020) | China;NS | NS;2 | 30 | 30 | 41.68±11.54 | | NS | NS | TG+Thalidomide | TG | 8 | ① |
| Lv(2020) | China;NS | NS;2 | 56 | 56 | 44.58±7.12 | 45.01±6.93 | 7.63±1.83 | 7.79±1.72 | TGP+Acitretin  +Compound flumethasone ointment | Acitretin+  Compound flumethasone ointment | 8 | ① |
| Huang et al.(2020) | China;NS | NS;2 | 56 | 56 | 42.21±2.16 | 42.13±2.21 | 5.37±1.15 | 5.42±1.14 | CG+Acitretin | Acitretin | 8 | ① |
| Chang et al.(2020) | China;NS | NS;2 | 33 | 30 | 66.42±5.05 | 66.83±5.51 | NS | NS | TGP+Halometasone+Calcipotriol | Halometasone+  Calcipotriol | 12 | ① |
| Cao(2020) | China;NS | NS;2 | 42 | 41 | 43.5±1.4 | 43.4±1.5 | NS | NS | CG+Acitretin | Acitretin | 6 | ① |
| Zhao(2019) | China;NS | NS;2 | 60 | 60 | 43.21±2.01 | 43.20±1.98 | NS | NS | TGP+Acitretin  +Compound flumethasone ointment | Acitretin+  Compound flumethasone ointment | 8 | ⑥ |
| Zhang(2019) | China;NS | NS;2 | 47 | 47 | 43.11±6.90 | | 5.29±1.44 | | TG+NB-UVB | NB-UVB | 8 | ① |
| Yin(2019) | China;NS | NS;2 | 36 | 36 | 44.0±6.6 | 44.8±6.3 | 5.1±2.3 | 5.7±2.4 | TGP+NB-UVB | NB-UVB | 12 | ① |
| Wu et al.(2019) | China;NS | NS;2 | 40 | 40 | 42.74±6.53 | 43.66±6.42 | 5.56±2.44 | 5.41±2.27 | TG+NB-UVB | NB-UVB | 12 | ①② |
| Tan and Tang(2019) | China;NS | NS;2 | 45 | 41 | 37.64±6.599 | 36.66±8.386 | 8.96±3.359 | 8.24±4.158 | TG+CG | Viaminate Capsules | 6 | ① |
| Peng(2019) | China;NS | NS;2 | 65 | 65 | 37.8±4.8 | 36.6±5.2 | 1.02±0.33 | 0.92±0.37 | CG+Fluticasone propionate | Fluticasone propionate | 6 | ①④⑥ |
| Lv(2019) | China;NS | NS;2 | 53 | 53 | 38.95±8.03 | 38.62±8.19 | 5.03±2.21 | 4.96±2.18 | CG+Acitretin | Acitretin | 6 | ① |
| Li(2019) | China;NS | NS;2 | 52 | 52 | 46.01±4.21 | 45.35±4.22 | 3.41±0.51 | 3.21±0.65 | CG+NB-UVB | NB-UVB | 12 | ②⑤ |
| Li and Li(2019) | China;NS | NS;2 | 48 | 48 | 58.47±7.24 | | 8.34±2.09 | | CG+NB-UVB | NB-UVB | 8 | ① |
| He et al.(2019) | China;hospital out/inpatients | NS;2 | 30 | 30 | 46.21±7.8 | 43.21±9.9 | 5.1±2.1 | 4.7±1.7 | TG+Urea ointment+Acupoint autohemotherapy | TG+Urea ointment | 8 | ① |
| Hao et al.(2019) | China;hospital inpatients | NS;2 | 40 | 40 | 35.5±9.8 | 35.0±10.5 | 14.7±3.0 | 15.4±4.8 | TGP+Acitretin  +NB-UVB | NB-UVB | 8 | ①③⑤⑥ |
| Zhang and Wang(2018) | China;NS | NS;2 | 50 | 50 | 44.12±5.77 | 44.19±5.80 | 6.74±1.43 | 6.62±1.40 | TGP+Acitretin  +NB-UVB | Acitretin+  NB-UVB | 8 | ①② |
| Yin et al.(2018) | China;NS | NS;2 | 50 | 50 | 37.83±10.29 | 37.29±9.37 | 3.77±1.47 | 3.92±1.57 | TG+Acitretin | Acitretin | 8 | ⑥ |
| Yan et al.(2018) | China;NS | NS;2 | 72 | 72 | 36.8±7.2 | 35.4±6.8 | 8.1±3.5 | 7.6±2.9 | CG+Acitretin  +NB-UVB | Acitretin+  NB-UVB | 8 | ① |
| Li et al.(2018) | China;hospital out/inpatients | NS;2 | 43 | 43 | 40.51±10.46 | 40.38±10.24 | 8.33±3.20 | 8.29±3.17 | CG+Acitretin | Acitretin | 6 | ①⑥ |
| Hu(2018) | China;NS | NS;2 | 20 | 20 | 45.50±7.05 | 43.50±7.55 | 0.46±0.13 | 0.33±0.09 | TG+Acitretin | Acitretin | 8 | ③⑤ |
| Wang(2017) | China;NS | NS;2 | 40 | 40 | 39.28±4.51 | 39.89±5.03 | 2.04±0.35 | 2.10±0.39 | CG+TG | CG | 8 | ② |
| Song et al.(2017) | China;NS | NS;2 | 63 | 63 | 43.52±12.03 | 43.29±11.76 | 10.64±8.06 | 10.81±8.22 | TGP+Acitretin  +Compound flumethasone ointment | Acitretin+  Compound flumethasone ointment | 8 | ①③⑤⑥ |
| Ling(2017) | China;NS | NS;2 | 43 | 43 | 36.45±5.12 | 36.21±4.81 | 5.12±1.21 | 5.22±1.31 | CG+Acitretin | Acitretin | 6 | ①② |
| Lin(2017) | China;NS | NS;2 | 67 | 62 | 39.28±10.21 | 40.18±11.83 | 18.81±5.33 | 19.74±6.21 | TGP+NB-UVB  +Calcipotriol | NB-UVB  +Calcipotriol | 8 | ⑤⑥ |
| Li(2017) | China;hospital outpatients | NS;2 | 37 | 37 | 37.15±9.24 | 36.52±9.16 | 5.12±1.88 | 5.18±2.04 | TGP+NB-UVB | NB-UVB | 8 | ① |
| Jiao(2017) | China;NS | NS;2 | 52 | 52 | 47.5±4.6 | 47.6±4.5 | NS | NS | CG+Calcipotriol | Calcipotriol | 8 | ③④⑤ |
| Fu and Xie(2017) | China;NS | NS;2 | 48 | 48 | 39.2±8.7 | 38.6±8.4 | 3.71±0.85 | 3.64±0.87 | CG+Acitretin  +Dithranol | Acitretin+  Dithranol | 4 | ⑤ |
| Du(2017) | China;NS | NS;2 | 42 | 42 | 39.76±4.31 | 3.75±0.98 | 36.14±3.29 | 3.82±0.96 | CG+Acitretin  +Dithranol | Acitretin+  Dithranol | 5 | ⑤ |
| Cheng(2017) | China;NS | NS;2 | 27 | 26 | 38.4±5.8 | 38.2±5.3 | 5.6±2.4 | 5.7±2.5 | CG+Acitretin | Acitretin | 8 | ① |
| Cha(2017) | China;NS | NS;2 | 41 | 40 | 38.0±5.1 | 37.7±5.6 | 7.3±2.3 | 7.5±2.0 | CG+Compound flumethasone ointment | Compound flumethasone ointment | 4 | ① |
| Zhu(2016) | China;hospital outpatients | NS;2 | 50 | 50 | 35.5±10.4 | 37.5±9.8 | NS | NS | TGP+Compound amino-polypeptide tablets | TG | 6 | ① |
| Zhu et al.(2016) | China;hospital out/inpatients | NS;2 | 20 | 10 | 35.0±10.0 | 37.5±9.8 | NS | NS | TGP+Urea ointment | TG+Urea ointment | 6 | ① |
| Zhang et al.(2016) | China;NS | NS;2 | 48 | 48 | 36.8±5.3 | 36.2±5.1 | 13.6±3.9 | 12.4±3.5 | TGP+Methotrexate | Methotrexate | 12 | ①⑥ |
| Shi(2016) | China;NS | NS;2 | 75 | 75 | 28.1±1.2 | | 7.2±0.2 | | CG+Viaminate Capsules | Viaminate Capsules | 8 | ① |
| Fan(2016) | China;NS | NS;2 | 98 | 98 | 30.4±6.5 | 31.2±6.8 | 4.6±2.5 | 4.7±2.4 | TG+0.05% Tazarotene gel | 0.05% Tazarotene gel | 12 | ⑤⑥ |
| Shen and Ye(2016) | China;NS | NS;2 | 40 | 40 | 36±4 | 35±5 | 5.5±1.2 | 5.0±1.3 | CG+Acitretin | Acitretin | 6 | ①⑥ |
| Wu et al.(2015) | China;NS | NS;2 | 50 | 50 | 44.0±9.2 | 43.8±9.7 | 5.0±3.1 | 5.2±2.4 | CG+Calcipotriol | Calcipotriol | 8 | ③④⑤ |
| Zou et al.(2015) | China;NS | NS;3 | 25 | | 37.75±5.61 | | NS | | TGP+NB-UVB | | 12 | ① |
|  |  |  | 26 | |  |  | NS | | NB-UVB | |  |  |
|  |  |  | 25 | |  |  | NS | | TGP | |  |  |
| Tan(2015) | China;NS | NS;2 | 58 | 58 | 49.1±10.2 | 48.4±10.6 | 10.3±3.4 | 10.1±3.2 | CG+Calcipotriol | Calcipotriol | 8 | ① |
| Sun(2015) | China;NS | NS;2 | 41 | 40 | 31.5±8.6 | 32.3±8.9 | 0.80±0.23 | 0.76±0.24 | TGP+Compound flumethasone ointment | Compound flumethasone ointment | 8 | ①⑥ |
| Liu(2015) | China;NS | NS;2 | 55 | 55 | 31.2±6.4 | 32.1±5.8 | 0.20±0.10 | 0.22±0.12 | TG+Dithranol | Cyclosporine A+Dithranol | 12 | ①② |
| Li et al.(2015) | China;NS | NS;2 | 40 | 40 | 40.7±13.9 | 41.2±13.6 | 12.2±3.5 | 12.3±3.3 | CG+Tretinoin ointment+NB-UVB | Tretinoin ointment  +NB-UVB | 8 | ① |
| Zhang (2014) | China;NS | NS;2 | 60 | 60 | 37.1±9.6 | 35.6±8.9 | 6.3±5.0 | 5.7±4.2 | CG+Acitretin | Acitretin | 8-12 | ① |
| Yue et al.(2014) | China;NS | NS;2 | 40 | 40 | NS | NS | NS | NS | TG | Acitretin | 4 | ② |
| Wang(2014) | China;hospital outpatients | NS;2 | 60 | 60 | 37.4±2.9 | 38.6±3.1 | 5.8±1.2 | 5.6±1.5 | CG+Desonide | Desonide | 4 | ① |
| Jiang et al.(2014) | China;hospital out/inpatients | NS;2 | 35 | 34 | 33.9±12.4 | 34.4±13.7 | 5.4±2.3 | 5.7±2.5 | TGP+Acitretin  +Desonide | Acitretin+  Desonide | 12 | ① |
| He et al.(2014) | China;hospital out/inpatients | NS;2 | 78 | 76 | 38.4±9.5 | 37.9±11.2 | 21.5±4.5 | 19.4±4.7 | TGP+NB-UVB  +Calcipotriol | NB-UVB  +Calcipotriol | 8 | ① |
| Chen et al.(2014) | China;hospital out/inpatients | NS;2 | 28 | 27 | 40.12±13.23 | 42.35±12.25 | 6.35±3.24 | 6.86±4.03 | TGP+Acitretin  +NB-UVB | Acitretin+  NB-UVB | 8 | ① |
| Chen(2014) | China;NS | NS;2 | 46 | 46 | 29.15±1.71 | 28.97±1.85 | 8.40±1.75 | 8.62±1.52 | CG+Viaminate Capsules | Viaminate Capsules | 8 | ① |
| Cai et al.(2014) | China;hospital outpatients | NS;2 | 38 | 35 | 43.66±13.35 | 45.40±12.26 | NS | NS | TGP+Calcipotriol | Calcipotriol | 12 | ① |
| Zhang(2013) | China;hospital outpatients | NS;2 | 30 | 30 | 36.7±11.6 | 37.2±10.8 | 3.8±1.7 | 4.0±2.1 | TG+Acitretin | Acitretin | 8 | ② |
| Zhang et al.(2013) | China;hospital outpatients | NS;2 | 40 | 40 | 30.85±11.91 | 29.72±11.29 | 2.25 | 2.17 | TGP+Halcinonide | Halcinonide | 8 | ① |
| Yang et al.(2013) | China;hospital outpatients | NS;2 | 33 | 33 | 35.74±11.53 | 33.89±12.78 | 5.81±5.21 | 5.62±5.28 | TGP+NB-UVB | NB-UVB | 8 | ① |
| Mao et al.(2013) | China;hospital outpatients | NS;3 | 35 | | NS | | NS | | TGP+NB-UVB | | 6 | ① |
|  |  |  | 33 | | NS | | NS | | NB-UVB | |  |  |
|  |  |  | 30 | | NS | | NS | | TGP | |  |  |
| Jiang et al.(2013) | China;hospital out/inpatients | NS;2 | 38 | 24 | 34.33±16.45 | | 15.48±12.25 | | TGP+NB-UVB | NB-UVB | 6 | ① |
| Ma et al.(2012) | China;hospital outpatients | NS;2 | 40 | 38 | 38.35±11.77 | 37.74±14.61 | 6.04±3.96 | 6.09±3.88 | TGP+NB-UVB  +Halcinonide | NB-UVB  +Halcinonide | 8 | ① |
| Hu et al.(2012) | China;hospital out/inpatients | NS;2 | 50 | 50 | 37.3 | 35.8 | 11.6 | 12.3 | TGP+NB-UVB | NB-UVB | 6 | ①③⑥ |
| Zhou et al.(2011) | China;hospital out/inpatients | NS;2 | 47 | 41 | 30.5 | | 6.7 | | CG+Compound flumethasone ointment | Compound flumethasone ointment | 4 | ① |
| Yang(2011) | China;NS | NS;2 | 34 | 28 | 23.9 | | NS | | CG+Acitretin | Acitretin | 8 | ① |
| Wang and Luan(2010) | China;hospital outpatients | NS;2 | 60 | 60 | 32.6 | 36.5 | NS | NS | CG+NB-UVB  +Urea ointment | NB-UVB+  Urea ointment | 4 | ① |
| Ye et al.(2009) | China;hospital outpatients | NS;2 | 27 | 26 | 31.2±11.4 | | 7.5±5.1 | | CG+Viaminate Capsules | Viaminate Capsules | 8 | ① |
| Wei and Liao(2009) | China;hospital outpatients | NS;2 | 72 | 60 | 37.5 | | 4.9 | | CG+Acitretin | Acitretin | 8 | ① |
| He(2008) | China;hospital outpatients | NS;2 | 19 | 18 | 34.3±10.1 | 35.5±14.3 | NS | NS | TG+Diammonium glycyrrhizinate | Acitretin | 8 | ① |
| Luo and Chen(2006) | China;hospital outpatients | NS;2 | 51 | 46 | 38.15 | 40.22 | 1.47 | 1.31 | CG+NB-UVB | NB-UVB | 8 | ① |

## Relative effects of different on PASI

| **TG+TOP** |  |  |  |  |  |  |  |  |  |  |  |  |  |  |
| --- | --- | --- | --- | --- | --- | --- | --- | --- | --- | --- | --- | --- | --- | --- |
| -6.81 (-13.95,0.34) | **TG+AAH** |  |  |  |  |  |  |  |  |  |  |  |  |  |
| **-8.22 (-12.53,-3.91)** | -1.41 (-8.65,5.84) | **CG+TG** |  |  |  |  |  |  |  |  |  |  |  |  |
| **-10.54 (-13.59,-7.48)** | -3.73 (-10.30,2.85) | -2.32 (-5.60,0.96) | **TGP+NB-UVB** |  |  |  |  |  |  |  |  |  |  |  |
| **-10.57 (-13.65,-7.49)** | -3.76 (-10.36,2.85) | -2.35 (-5.69,0.99) | -0.03 (-1.40,1.34) | **TGP+TOP** |  |  |  |  |  |  |  |  |  |  |
| **-10.56 (-13.69,-7.44)** | -3.76 (-10.37,2.85) | -2.35 (-5.70,1.00) | -0.03 (-1.42,1.37) | 0.00 (-1.52,1.53) | **CG+TOP** |  |  |  |  |  |  |  |  |  |
| **-10.63 (-15.45,-5.80)** | -3.82 (-9.09,1.45) | -2.41 (-7.39,2.57) | -0.09 (-4.03,3.84) | -0.06 (-4.04,3.92) | -0.06 (-4.06,3.93) | **TG+CT** |  |  |  |  |  |  |  |  |
| **-10.97 (-14.03,-7.91)** | -4.16 (-10.74,2.42) | -2.76 (-6.04,0.53) | -0.44 (-1.68,0.81) | -0.41 (-1.79,0.98) | -0.41 (-1.82,1.00) | -0.34 (-4.29,3.60) | **CG+CT** |  |  |  |  |  |  |  |
| **-11.03 (-14.42,-7.65)** | -4.22 (-10.96,2.51) | -2.82 (-6.41,0.78) | -0.50 (-2.41,1.42) | -0.47 (-2.48,1.55) | -0.47 (-2.50,1.56) | -0.40 (-4.61,3.80) | -0.06 (-1.99,1.87) | **TG+NB-UVB** |  |  |  |  |  |  |
| **-11.87 (-17.62,-6.12)** | **-5.06 (-9.30,-0.82)** | -3.65 (-9.53,2.22) | -1.33 (-6.36,3.69) | -1.30 (-6.36,3.76) | -1.30 (-6.37,3.76) | -1.24 (-4.36,1.88) | -0.90 (-5.92,4.13) | -0.84 (-6.07,4.40) | **TG** |  |  |  |  |  |
| **-11.80 (-14.98,-8.63)** | -5.00 (-11.63,1.64) | **-3.59 (-6.98,-0.20)** | -1.27 (-2.77,0.23) | -1.24 (-2.85,0.38) | -1.24 (-2.88,0.40) | -1.18 (-5.21,2.85) | -0.83 (-2.28,0.62) | -0.77 (-2.87,1.33) | 0.06 (-5.03,5.16) | **CG+NB-UVB** |  |  |  |  |
| **-13.96 (-16.88,-11.03)** | **-7.15 (-13.67,-0.63)** | **-5.74 (-8.91,-2.57)** | **-3.42 (-4.29,-2.55)** | **-3.39 (-4.45,-2.33)** | **-3.39 (-4.49,-2.30)** | -3.33 (-7.17,0.51) | **-2.98 (-3.87,-2.09)** | **-2.92 (-4.63,-1.21)** | -2.09 (-7.04,2.86) | **-2.15 (-3.37,-0.93)** | **CT** |  |  |  |
| **-14.45 (-17.75,-11.15)** | **-7.64 (-14.33,-0.94)** | **-6.23 (-9.75,-2.71)** | **-3.91 (-5.44,-2.38)** | **-3.88 (-5.74,-2.02)** | **-3.88 (-5.76,-2.00)** | -3.82 (-7.95,0.32) | **-3.47 (-5.25,-1.70)** | **-3.41 (-5.71,-1.12)** | -2.58 (-7.76,2.60) | **-2.64 (-4.60,-0.68)** | -0.49 (-2.02,1.04) | **TGP** |  |  |
| **-14.49 (-17.96,-11.02)** | **-7.68 (-14.47,-0.90)** | **-6.27 (-9.96,-2.59)** | **-3.95 (-6.03,-1.88)** | **-3.92 (-6.09,-1.76)** | **-3.93 (-6.10,-1.75)** | -3.86 (-8.14,0.42) | **-3.52 (-5.31,-1.72)** | **-3.46 (-6.00,-0.91)** | -2.62 (-7.92,2.67) | **-2.68 (-4.68,-0.69)** | -0.53 (-2.42,1.35) | -0.04 (-2.47,2.38) | **CG** |  |
| **-16.13 (-24.14,-8.11)** | **-9.32 (-16.34,-2.30)** | -7.91 (-16.02,0.20) | -5.59 (-13.11,1.92) | -5.56 (-13.10,1.98) | -5.56 (-13.11,1.98) | -5.50 (-11.90,0.90) | -5.16 (-12.67,2.36) | -5.10 (-12.75,2.56) | -4.26 (-9.85,1.33) | -4.32 (-11.89,3.24) | -2.17 (-9.64,5.29) | -1.68 (-9.30,5.94) | -1.64 (-9.34,6.06) | **TGP+CT** |

## Relative effects of different on CD4^+^/CD8^+^

| **CG+CT** |  |  |  |  |  |  |  |  |  |
| --- | --- | --- | --- | --- | --- | --- | --- | --- | --- |
| 0.05 (-0.32,0.42) | **TGP+TOP** |  |  |  |  |  |  |  |  |
| **0.23 (0.03,0.43)** | 0.18 (-0.17,0.53) | **TG+CT** |  |  |  |  |  |  |  |
| 0.24 (-0.01,0.49) | 0.19 (-0.19,0.57) | 0.01 (-0.21,0.23) | **CG+NB-UVB** |  |  |  |  |  |  |
| **0.26 (0.03,0.49)** | 0.21 (-0.16,0.58) | 0.03 (-0.18,0.23) | 0.02 (-0.23,0.27) | **TG+NB-UVB** |  |  |  |  |  |
| **0.29 (0.09,0.50)** | 0.24 (-0.11,0.60) | 0.06 (-0.11,0.24) | 0.05 (-0.17,0.28) | 0.04 (-0.17,0.24) | **TG+TOP** |  |  |  |  |
| **0.32 (0.06,0.58)** | 0.27 (-0.12,0.66) | 0.09 (-0.15,0.33) | 0.08 (-0.20,0.36) | 0.06 (-0.20,0.33) | 0.03 (-0.22,0.27) | **TGP+NB-UVB** |  |  |  |
| **0.47 (0.12,0.82)** | 0.42 (-0.03,0.87) | 0.24 (-0.09,0.57) | 0.23 (-0.02,0.48) | 0.21 (-0.14,0.56) | 0.18 (-0.16,0.51) | 0.15 (-0.22,0.52) | **CG+TG** |  |  |
| **0.58 (0.42,0.74)** | **0.53 (0.20,0.86)** | **0.35 (0.23,0.47)** | **0.34 (0.15,0.53)** | **0.32 (0.16,0.49)** | **0.29 (0.16,0.41)** | **0.26 (0.05,0.47)** | 0.11 (-0.20,0.42) | **CT** |  |
| **0.76 (0.46,1.06)** | **0.71 (0.29,1.13)** | **0.53 (0.25,0.81)** | **0.52 (0.35,0.69)** | **0.50 (0.20,0.81)** | **0.47 (0.18,0.75)** | **0.44 (0.11,0.77)** | **0.29 (0.12,0.46)** | 0.18 (-0.07,0.43) | **CG** |

## Relative effects of different on IL-23

| **CG+TOP** |  |  |  |  |  |  |
| --- | --- | --- | --- | --- | --- | --- |
| -8.38 (-25.19,8.42) | **CG+NB-UVB** |  |  |  |  |  |
| **-19.79 (-31.98,-7.59)** | -11.40 (-29.29,6.49) | **TGP+TOP** |  |  |  |  |
| **-30.72 (-39.75,-21.69)** | **-22.33 (-38.24,-6.43)** | **-10.93 (-21.48,-0.39)** | **TGP+NB-UVB** |  |  |  |
| **-39.38 (-46.83,-31.93)** | **-31.00 (-46.06,-15.94)** | **-19.60 (-29.25,-9.95)** | **-8.67 (-13.76,-3.57)** | **CT** |  |  |
| **-49.54 (-60.38,-38.69)** | **-41.15 (-58.15,-24.15)** | **-29.75 (-42.04,-17.46)** | **-18.82 (-26.69,-10.95)** | **-10.15 (-18.03,-2.28)** | **TGP** |  |
| **-99.14 (-123.43,-74.86)** | **-90.76 (-118.35,-63.17)** | **-79.36 (-104.40,-54.31)** | **-68.43 (-92.09,-44.76)** | **-59.76 (-82.87,-36.65)** | **-49.61 (-74.02,-25.19)** | **TG+CT** |

## Relative effects of different on IL-17

| **CG+NB-UVB** |  |  |  |  |  |  |  |  |  |  |
| --- | --- | --- | --- | --- | --- | --- | --- | --- | --- | --- |
| -3.91 (-24.61,16.79) | **CG** |  |  |  |  |  |  |  |  |  |
| -9.97 (-42.37,22.42) | -6.07 (-44.50,32.37) | **TG+CT** |  |  |  |  |  |  |  |  |
| -16.94 (-36.90,3.02) | -13.03 (-41.78,15.71) | -6.97 (-37.28,23.34) | **TGP+TOP** |  |  |  |  |  |  |  |
| -17.11 (-36.42,2.20) | -13.20 (-41.50,15.10) | -7.14 (-37.03,22.76) | -0.17 (-15.71,15.38) | **CG+TOP** |  |  |  |  |  |  |
| -17.73 (-38.72,3.26) | -13.82 (-43.29,15.65) | -7.76 (-38.76,23.25) | -0.79 (-18.35,16.78) | -0.62 (-17.50,16.26) | **TGP+NB-UVB** |  |  |  |  |  |
| -19.24 (-45.61,7.13) | -15.34 (-48.85,18.18) | -9.27 (-44.14,25.60) | -2.30 (-26.07,21.47) | -2.13 (-25.36,21.09) | -1.51 (-26.16,23.13) | **TG+TOP** |  |  |  |  |
| -26.09 (-52.42,0.23) | -22.19 (-55.66,11.29) | -16.12 (-50.96,18.72) | -9.15 (-32.86,14.56) | -8.98 (-32.16,14.19) | -8.36 (-32.96,16.23) | -6.85 (-36.17,22.47) | **CG+CT** |  |  |  |
| **-27.77 (-49.60,-5.93)** | -23.86 (-53.93,6.22) | -17.79 (-49.37,13.79) | -10.82 (-29.43,7.78) | -10.66 (-28.57,7.26) | -10.04 (-29.75,9.68) | -8.52 (-33.88,16.84) | -1.67 (-26.99,23.64) | **TG+NB-UVB** |  |  |
| **-28.87 (-52.04,-5.71)** | -24.97 (-56.02,6.09) | -18.90 (-51.42,13.62) | -11.93 (-32.06,8.20) | -11.76 (-31.28,7.75) | -11.14 (-27.64,5.35) | -9.63 (-36.15,16.89) | -2.78 (-29.26,23.69) | -1.11 (-23.12,20.91) | **TGP** |  |
| **-29.99 (-46.25,-13.74)** | -26.09 (-52.40,0.22) | -20.02 (-48.04,8.00) | **-13.05 (-24.62,-1.48)** | **-12.88 (-23.30,-2.47)** | -12.26 (-25.55,1.02) | -10.75 (-31.51,10.01) | -3.90 (-24.60,16.80) | -2.23 (-16.80,12.34) | -1.12 (-17.62,15.38) | **CT** |

## Relative effects of different on TNF-α

| **TGP+TOP** |  |  |  |  |  |  |  |  |  |
| --- | --- | --- | --- | --- | --- | --- | --- | --- | --- |
| -9.41 (-25.05,6.22) | **TGP+NB-UVB** |  |  |  |  |  |  |  |  |
| -9.37 (-29.52,10.77) | 0.04 (-21.90,21.98) | **TG+TOP** |  |  |  |  |  |  |  |
| -13.10 (-33.31,7.10) | -3.69 (-25.68,18.30) | -3.73 (-29.12,21.66) | **CG+NB-UVB** |  |  |  |  |  |  |
| **-14.14 (-28.00,-0.28)** | -4.73 (-21.09,11.63) | -4.77 (-25.47,15.94) | -1.04 (-21.80,19.72) | **CG+CT** |  |  |  |  |  |
| -14.82 (-41.77,12.12) | -5.41 (-33.72,22.90) | -5.45 (-36.47,25.57) | -1.72 (-19.62,16.18) | -0.68 (-28.04,26.68) | **CG** |  |  |  |  |
| -15.43 (-31.11,0.26) | -6.01 (-23.95,11.92) | -6.05 (-28.03,15.92) | -2.32 (-24.35,19.70) | -1.29 (-17.69,15.11) | -0.60 (-28.93,27.73) | **CG+TOP** |  |  |  |
| **-16.18 (-31.83,-0.52)** | -6.76 (-24.67,11.15) | -6.80 (-28.75,15.15) | -3.07 (-25.07,18.93) | -2.03 (-18.41,14.34) | -1.35 (-29.67,26.96) | -0.75 (-18.69,17.20) | **TG+NB-UVB** |  |  |
| -18.20 (-38.31,1.91) | -8.79 (-30.70,13.12) | -8.83 (-34.15,16.49) | -5.10 (-30.46,20.26) | -4.06 (-24.73,16.61) | -3.38 (-34.38,27.62) | -2.78 (-24.71,19.16) | -2.03 (-23.95,19.89) | **TG+CT** |  |
| **-18.54 (-27.74,-9.35)** | -9.13 (-21.78,3.53) | -9.17 (-27.09,8.75) | -5.44 (-23.42,12.55) | -4.40 (-14.77,5.97) | -3.72 (-29.04,21.60) | -3.12 (-15.82,9.59) | -2.37 (-15.04,10.30) | -0.34 (-18.22,17.54) | **CT** |

## Safety evaluation

| **Study ID** | **Sample size（n）** | | **Intervention characteristics** | | **Adverse event** | |
| --- | --- | --- | --- | --- | --- | --- |
|  | **T** | **C** | **T** | **C** | **T** | **C** |
| Wang and Wang(2024) | 35 | 35 | TG+NB-UVB | NB-UVB | 1 white blood cell damage, 1 liver function impairment, 2 rashes, 1 diarrhea and nausea | 2 white blood cell injuries, 1 rash, 1 blister, 2 diarrhea and nausea |
| Ma and Zhu(2024) | 41 | 41 | TG+NB-UVB | NB-UVB | 0 | 0 |
| Zhang(2022) | 56 | 56 | CG+NB-UVB | CG | NS | NS |
| Wu(2022) | 33 | 30 | TGP+TOP | TOP | NS | NS |
| Wang(2022) | 64 | 64 | CG+Acitretin | Acitretin | 1 itch of skin, 1 dry skin | 3 itch of skin, 3 dry skin, 2 erythema |
| Liu(2022) | 30 | 30 | CG+Acitretin | Acitretin | 1 elevated blood lipids, 1 dry skin | 1 elevated blood lipids, 1 dry skin, 1 headache |
| Lin(2022) | 47 | 47 | TGP+Acitretin+Dithranol | Acitretin+Dithranol | 3 dry skin, 1 diarrhoea, 1 itch of skin, 2 desquamation | 2 dry skin, 2 diarrhoea, 2 itch of skin, 3 dyslipidemia, 2 1iver damage, 1 desquamation |
| Wei(2022) | 48 | 48 | CG+NB-UVB | NB-UVB | NS | NS |
| Zhang(2021) | 52 | 51 | CG+Acitretin | Acitretin | 3 dry eyes, 2 mouth parched and tongue scorched, 2 itch of skin, 1 liver and kidney function impairment | 6 dry eyes, 4 mouth parched and tongue scorched, 4 itch of skin, 7 liver and kidney function impairment |
| Wang et al.(2021) | 30 | 30 | TG+NB-UVB | NB-UVB | 2 nausea and vomiting, 1 diarrhea and abdominal pain, 1 rash, 1 abnormal blood counts | 1 nausea and vomiting, 1 diarrhea and abdominal pain, 1 rash, 1 abnormal blood counts |
| Wang and Li(2021) | 43 | 43 | CG+Fluticasone propionate | Fluticasone propionate | NS | NS |
| Ren and Zhao(2021) | 30 | | TGP+NB-UVB | | NS | NS |
|  | 30 | | TGP | | NS | NS |
|  | 30 | | NB-UVB | | NS | NS |
| Peng(2021) | 42 | 42 | TG+Acitretin+Thalidomide | Acitretin+Thalidomide | 1 nausea, 1 fatigue | 2 fatigue, 1 drowsiness |
| Ma et al.(2021) | 39 | 39 | CG+NB-UVB+Halometasone+Calcipotriol | NB-UVB+Halometasone+ Calcipotriol | NS | NS |
| Dong(2021) | 33 | 33 | CG+Acitretin | Acitretin | NS | NS |
| Zhai et al.(2021) | 40 | 40 | TGP+Acitretin+Budesonide | Acitretin+Budesonide | 0 | 0 |
| Zhuo et al.(2020) | 50 | 50 | CG+Tacrolimus+Ebastine | CG+Tacrolimus | NS | NS |
| Zhang and Song(2020) | 40 | 40 | CG+Acitretin | CG | 1 itch of skin, 2 dry skin, 1 erythema | 2 itch of skin, 2 dry skin, 1 erythema |
| Shang et al.(2020) | 30 | 30 | TGP+Desonide | Desonide | 1 mild abdominal discomfort | 0 |
| Ren et al.(2020) | 30 | 30 | TG+Thalidomide | TG | 5 mild elevation of liver enzymes | 4 mild elevation of liver enzymes |
| Lv(2020) | 56 | 56 | TGP+Acitretin+Compound flumethasone ointment | Acitretin+Compound flumethasone ointment | 6 dry mouth/eyes/skin, gastrointestinal discomfort | 5dry mouth/eyes/skin, gastrointestinal discomfort |
| Huang et al.(2020) | 56 | 56 | CG+Acitretin | Acitretin | 1 dry mouth, 1 dry eyes, 1 dry skin | 4 dry mouth, 2 dry eyes, 1 dry skin, 3 hair loss, 1 elevated blood lipids |
| Chang et al.(2020) | 33 | 30 | TGP+Halometasone  +Calcipotriol | Halometasone+Calcipotriol | 3 diarrhoea | 1 worsening skin lesions |
| Cao(2020) | 42 | 41 | CG+Acitretin | Acitretin | 2 itch of skin | 1 itch of skin,1 dry lips,1 erythema |
| Zhao(2019) | 60 | 60 | TGP+Acitretin+Compound flumethasone ointment | Acitretin+Compound flumethasone ointment | 4 gastrointestinal reactions,1 flushed face,1 headache | 3 gastrointestinal reactions,1 dizzy |
| Zhang(2019) | 47 | 47 | TG+NB-UVB | NB-UVB | NS | NS |
| Yin(2019) | 36 | 36 | TGP+NB-UVB | NB-UVB | NS | NS |
| Wu et al.(2019) | 40 | 40 | TG+NB-UVB | NB-UVB | 0 | 0 |
| Tan and Tang(2019) | 45 | 41 | TG+CG | Viaminate Capsules | NS | NS |
| Peng(2019) | 65 | 65 | CG+Fluticasone propionate | Fluticasone propionate | NS | NS |
| Lv(2019) | 53 | 53 | CG+Acitretin | Acitretin | 3 itch of skin, 4 dry skin, | 1 erythema, 4 itch of skin, 5 dry skin |
| Li(2019) | 52 | 52 | CG+NB-UVB | NB-UVB | 1 gastrointestinal reaction, 1 mild itching, 1 mild burning sensation | 2 gastrointestinal reaction, 3 mild itching, 2 mild burning sensation |
| Li and Li(2019) | 48 | 48 | CG+NB-UVB | NB-UVB | 2 dry and itchy skin,1 elevation of blood pressure, 1 blister, 1 erythema | 4 dry and itchy skin, 2 blister, 2 erythema |
| He et al.(2019) | 30 | 30 | TG+Urea ointment+Acupoint autohemotherapy | TG+Urea ointment | 1 gastrointestinal symptom | 2 gastrointestinal symptom |
| Hao et al.(2019) | 40 | 40 | TGP+Acitretin+NB-UVB | NB-UVB | 2 symptoms unspecified | 14 symptoms unspecified |
| Zhang and Wang(2018) | 50 | 50 | TGP+Acitretin+NB-UVB | Acitretin+NB-UVB | NS | NS |
| Yin et al.(2018) | 50 | 50 | TG+Acitretin | Acitretin | NS | NS |
| Yan et al.(2018) | 72 | 72 | CG+Acitretin+NB-UVB | Acitretin+NB-UVB | 51 dry lips, 27 dry and itchy skin, 3 mild elevation of alanine aminotransferase, 3 mildly elevated blood lipid levels | 58 dry lips, 34 dry and itchy skin, 10 mild elevation of alanine aminotransferase, 7 mildly elevated blood lipid levels |
| Li et al.(2018) | 43 | 43 | CG+Acitretin | Acitretin | 3 itch of skin, 5 dry skin, 10 dry lips | 5 itch of skin, 6 dry skin, 2 erythema, 12 dry lips |
| Hu(2018) | 20 | 20 | TG+Acitretin | Acitretin | 1 dry mouth and loss of appetite; 1 abdominal pain, lip fissure and nausea | 4 dry mouth and loss of appetite; 5 abdominal pain, lip fissure and nausea |
| Wang(2017) | 40 | 40 | CG+TG | CG | 0 | 0 |
| Song et al.(2017) | 63 | 63 | TGP+Acitretin+Compound flumethasone ointment | Acitretin+Compound flumethasone ointment | 7 dry mouth/eyes/ skin, gastrointestinal reactions | 6 dry mouth/eyes /skin, gastrointestinal reactions |
| Ling(2017) | 43 | 43 | CG+Acitretin | Acitretin | 2 itch of skin, 3 dry skin,  5 dry lips | 2 erythema,3 itch of skin,  4 dry skin,6 dry lips |
| Lin(2017) | 67 | 62 | TGP+NB-UVB+Calcipotriol | NB-UVB+Calcipotriol | NS | NS |
| Li(2017) | 37 | 37 | TGP+NB-UVB | NB-UVB | 3 abdominal discomfort and mild diarrhea | 2 mild pruritus and dryness of the skin |
| Jiao(2017) | 52 | 52 | CG+Calcipotriol | Calcipotriol | NS | NS |
| Fu and Xie(2017) | 48 | 48 | CG+Acitretin+Dithranol | Acitretin+Dithranol | 2 abdominal pain, 1 dizzy, 1 mild decrease in serum potassium, 1 mild elevation of transaminases | 2 headache, 1 mildly elevated blood lipid levels, 1 vomit |
| Du(2017) | 42 | 42 | CG+Acitretin+Dithranol | Acitretin+Dithranol | NS | NS |
| Cheng(2017) | 27 | 26 | CG+Acitretin | Acitretin | 4 dry eyes, mouth and skin | 6 dry eyes, mouth and skin; 1 alopecia; 1 elevated blood lipids |
| Cha(2017) | 41 | 40 | CG+Compound flumethasone ointment | Compound flumethasone ointment | NS | NS |
| Zhu(2016) | 50 | 50 | TGP+Compound amino-polypeptide tablets | TG | 4 common cold, 2 pruritus,  2 dry mouth and desquamation,  1 loss of appetite | 3 common cold, 2 diarrhoea,  4 loss of appetite |
| Zhu et al.(2016) | 20 | 10 | TGP+Urea ointment | TG+Urea ointment | 3 common cold, 1 diarrhea | 1 common cold, 1 loss of appetite |
| Zhang et al.(2016) | 48 | 48 | TGP+Methotrexate | Methotrexate | 1 liver function impairment,6 gastrointestinal discomfort | 3 dental ulcer, 6 liver function impairment, 2 mild leukopenia, 3 gastrointestinal discomfort |
| Shi(2016) | 75 | 75 | CG+Viaminate Capsules | Viaminate Capsules | 1 abnormal blood routine,  2 liver function impairment,  1 unspecified | 2 abnormal urinalysis, 1 abnormal blood routine, 1 liver function impairment, 1 unspecified |
| Fan(2016) | 98 | 98 | TG+0.05% Tazarotene gel | 0.05% Tazarotene gel | NS | NS |
| Shen and Ye(2016) | 40 | 40 | CG+Acitretin | Acitretin | 9 dry lips, 5 dry skin,  3 itch of skin | 11 dry lips, 6 dry skin,  5 itch of skin, 2 erythema |
| Wu et al.(2015) | 50 | 50 | CG+Calcipotriol | Calcipotriol | NS | NS |
| Zou et al.(2015) | 25 | | TGP+NB-UVB | | 5 dry eyes/mouth/skin and itching,2 nausea | |
|  | 26 | | NB-UVB | | 8 pruritus and desquamation | |
|  | 25 | | TGP | | 3 nausea, 3 diarrhoea, 1 mild elevation of alanine aminotransferase | |
| Tan(2015) | 58 | 58 | CG+Calcipotriol | Calcipotriol | 3 mild skin irritation, 2 mild gastrointestinal reactions | 4 mild skin irritation |
| Sun(2015) | 41 | 40 | TGP+Compound flumethasone ointment | Compound flumethasone ointment | 3 Mild elevation of glutamic and aspartic transaminases, 1 mild abdominal pain, 2 mild diarrhea | 1 mild diarrhea |
| Liu(2015) | 55 | 55 | TG+Dithranol | Cyclosporine A+Dithranol | 1 vomiting, 2 diarrhea, 2 menstrual disorders | 4 transient increases in serum creatinine and blood urea nitrogen, 2 nausea and vomiting, 1 diarrhea, 1 rash |
| Li et al.(2015) | 40 | 40 | CG+Tretinoin ointment+NB-UVB | Tretinoin ointment +NB-UVB | 7 skin mucosal reaction,  3 elevated triglycerides | 14 skin mucosal reaction,  5 elevated triglycerides,  1 elevated transaminase levels |
| Zhang (2014) | 60 | 60 | CG+Acitretin | Acitretin | (Specific numbers NS) Dry skin and lips,  desquamation, swelling, erythema, pruritus | |
| Yue et al.(2014) | 40 | 40 | TG | Acitretin | NS | NS |
| Wang(2014) | 60 | 60 | CG+Desonide | Desonide | 1 dry skin | 1 mild erythema |
| Jiang et al.(2014) | 35 | 34 | TGP+Acitretin+Desonide | Acitretin+Desonide | 16 dryness and itch of skin,  15 cheilosis, 10 dryness of eye,  2 loose stools, 3 rhinorrhagia,  1 hyperlipemia | 25 dryness and itch of skin, 23 cheilosis, 18 dryness of eye, 5 rhinorrhagia, 6 dysfunction of liver, 8 hyperlipemia |
| He et al.(2014) | 78 | 76 | TGP+NB-UVB+Calcipotriol | NB-UVB+Calcipotriol | 2 mild diarrhea | 3 skin lesions are red and pricking |
| Chen et al.(2014) | 28 | 27 | TGP+Acitretin+NB-UVB | Acitretin+NB-UVB | 5 diarrhea or abdominal discomfort, 3 mild burning sensation and itching of skin, 4 chapped lips, 2 elevated alanine aminotransferase | 4 mild burning sensation and itching of skin, 3 chapped lips, 2 elevated alanine aminotransferase |
| Chen(2014) | 46 | 46 | CG+Viaminate Capsules | Viaminate Capsules | 2 abnormal blood routine,  1 liver function impairment,  2 unspecified | 1 abnormal blood routine,  1 abnormal urinalysis,  1 liver function impairment,  1 unspecified |
| Cai et al.(2014) | 38 | 35 | TGP+Calcipotriol | Calcipotriol | 1 diarrhoea, 2 Gastrointestinal discomfort | 0 |
| Zhang(2013) | 30 | 30 | TG+Acitretin | Acitretin | 16 dry and scaly skin and mucous membranes, 2 gastrointestinal discomfort | 20 dry and scaly skin and mucous membranes, 6 gastrointestinal discomfort, 2 itchy skin, 1 mild elevation of triglycerides and alanine aminotransferase |
| Zhang et al.(2013) | 40 | 40 | TGP+Halcinonide | Halcinonide | 3 diarrhea and abdominal pain,  1 stomachache | 0 |
| Yang et al.(2013) | 33 | 33 | TGP+NB-UVB | NB-UVB | 5 diarrhoea, 3 dry skin with pruritus, 2 light red macule | 4 dry skin with pruritus,  1 light red macule |
| Mao et al.(2013) | 35 | | TGP+NB-UVB | | 4 mild diarrhea, 6dry skin, desquamation, pruritus, erythema | |
|  | 33 | | NB-UVB | | 5 dry skin, desquamation, pruritus, erythema | |
|  | 30 | | TGP | | 3 mild diarrhea | |
| Jiang et al.(2013) | 38 | 24 | TGP+NB-UVB | NB-UVB | 2 itchy skin, 2 dry skin,  1 erythema | 3 itchy skin, 4 dry skin,  2 erythema |
| Ma et al.(2012) | 40 | 38 | TGP+NB-UVB+Halcinonide | NB-UVB+Halcinonide | 3 mild diarrhea, 1 mild burning sensation and pruritus of skin | 5 mild burning sensation and pruritus of skin |
| Hu et al.(2012) | 50 | 50 | TGP+NB-UVB | NB-UVB | (Specific numbers NS) Dry skin, pruritus, erythema | |
| Zhou et al.(2011) | 47 | 41 | CG+Compound flumethasone ointment | Compound flumethasone ointment | 3 mildly elevated blood pressure, 3 local skin redness and pruritus | 4 local skin redness and pruritus |
| Yang(2011) | 34 | 28 | CG+Acitretin | Acitretin | 22 dry skin, 3 cheilitis/cracked lips, 2 erythema/pruritus, 1 elevated alanine aminotransferase, 3 hyperlipidemia | 28 dry skin, 7 cheilitis/cracked lips, 7 erythema/pruritus,  5 elevated alanine aminotransferase,  4 hyperlipidemia |
| Wang and Luan(2010) | 60 | 60 | CG+NB-UVB  +Urea ointment | NB-UVB+Urea ointment | 2 mild dizziness and fatigue,  14 burning sensation/ pruritus/erythema | 20 burning sensation/ pruritus/erythema |
| Ye et al.(2009) | 27 | 26 | CG+Viaminate Capsules | Viaminate Capsules | 3 elevated blood lipids | 4 elevated blood lipids, 4 mild elevation of transaminases |
| Wei and Liao(2009) | 72 | 60 | CG+Acitretin | Acitretin | 31 dryness (mucocutaneous), 6 Hyperlipidemia, 2 Elevated alanine aminotransferase,  2 headache | 42 dryness (mucocutaneous), 5 alopecia, 10 elevated alanine aminotransferase, 3 hyperlipidemia, 1 headache |
| He(2008) | 19 | 18 | TG+Diammonium glycyrrhizinate | Acitretin | 1 abnormal blood counts, 2 irregular menstruation | 12 dry lips; 5 dry, itchy skin; 2 dry oral mucosa; 7 dry eyes;1 hair loss |
| Luo and Chen(2006) | 51 | 46 | CG+NB-UVB | NB-UVB | 3 erythema | 8 erythema |

# Supplementary Figures

**3.1 Summary of the risk of bias in each study**

**
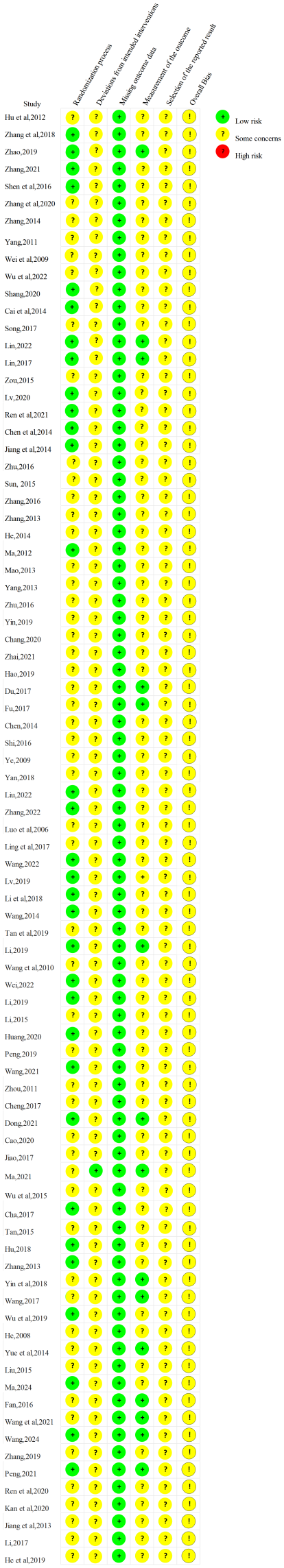
**
